# Supplementary material for: Achieving ultrahigh instantaneous power density of 10 MW/m2 by leveraging the opposite-charge-enhanced transistor-like triboelectric nanogenerator (OCT-TENG)
Source: Nat Commun. 2021 Sep 15;12:5470. doi: 10.1038/s41467-021-25753-7 (PMC8443631; doi:10.1038/s41467-021-25753-7)
Supplement: Supplementary file 1 — Supplementary Information [file 41467_2021_25753_MOESM1_ESM.pdf]

Supplementary Information

Achieving Ultrahigh Instantaneous Power Density of 10 MW/m^2 by Leveraging the Opposite-Charge-Enhanced Transistor-Like Triboelectric Nanogenerator (OCT-TENG)

Hao Wu [1,2], Steven Wang[2], Zuankai Wang [2,*], Yunlong Zi [1,*]

[1] Department of Mechanical and Automation Engineering, The Chinese University of Hong Kong, Shatin, New Territories, Hong Kong, China

[2] Department of Mechanical Engineering, City University of Hong Kong, Hong Kong, China

[*] Corresponding emails: zuanwang@cityu.edu.hk; ylzi@cuhk.edu.hk

Table of Contents

I. Comparison of the OCT-TENG, the SFT-TENG (Control #1), and the TENG-UDS (Control #2) (Supplementary Figures 1-6)

II. The current generation (load resistance of 22 Ω) at Stage 1 (Supplementary Figure 7)

III. Comparison of the power density of OCT-TENG compared to previous reports (Supplementary Figure 8)

IV. Equivalent circuit (Supplementary Figure 9)

V. Details of charge and current output of OCT-TENG (Supplementary Figure 10)

VI. Capacitor charging results of OCT-TENG and Control TENG (Supplementary Figures 11-13)

VII. Peak current depending on load resistance (Supplementary Figure14)

VIII. Photograph of operation of OCT-TENG (Supplementary Figure15)

IX. The U-Q plot of the OCT-TENG (varying load resistance) (Supplementary Figure 16)

X. Experiments based on varying parameters (including the effective area, the distance between E_L and E_R, and the shape of the electrode on the stator) (Supplementary Figures 17-20)

XI. Schematic of the "four-ports" method and experimental results (Supplementary Figures 21-24)

XII. Tribo-charges generation on ternary materials (Supplementary Figure 25)

XIII. The stability and durability of OCT-TENG (Supplementary Figures 26-28)

XIV. Powering watch and thermometer using OCT-TENG (Supplementary Figure 29)

XV. The energy output of the OCT-TENG (direct and wireless) (Supplementary Figure 30)

XVI. The current measurement circuit for the U-Q curve (Supplementary Figure 31)

Supplementary Note 1. Calculation of the charge transfer in OCT-TENG

(Supplementary Figures 32-37)

I. Comparison of the OCT-TENG, the SFT-TENG (Control #1), and the TENG-UDS (Control #2) (Supplementary Figures 1-6)

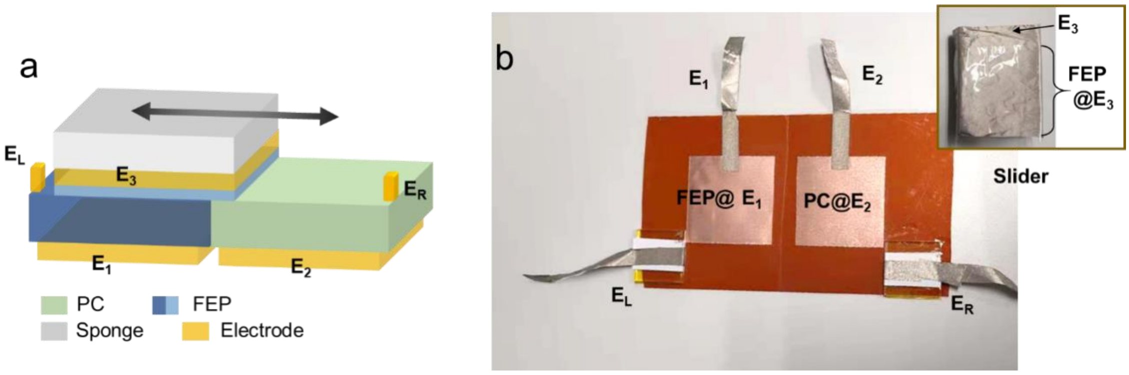

Supplementary Figure 1. (a) Schematic of the architecture of the OCT-TENG. **(b)** Photograph of the OCT-TENG.

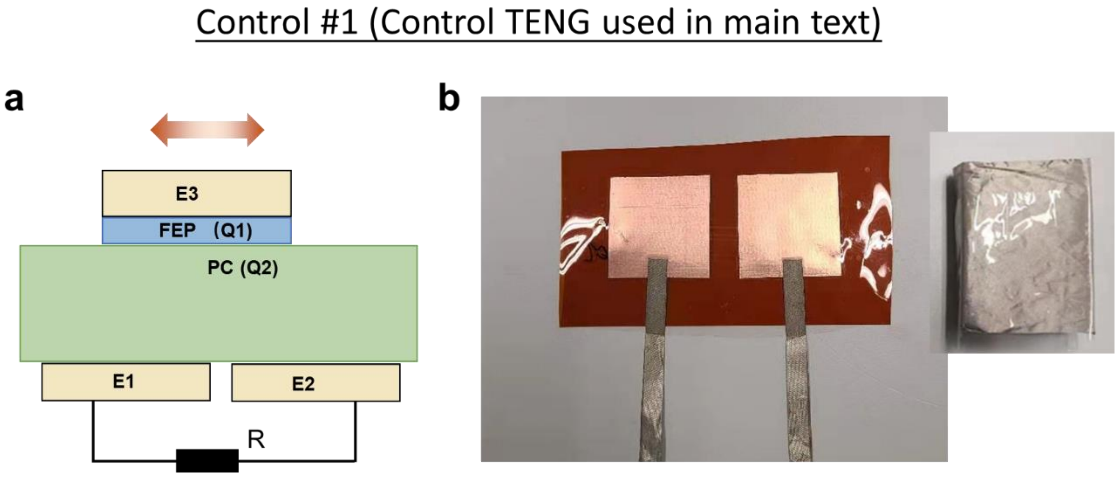

Supplementary Figure 2. (a) Schematic and **(b)** the photograph of the sliding-freestanding triboelectric nanogenerator (SFT-TENG). (Control #1)

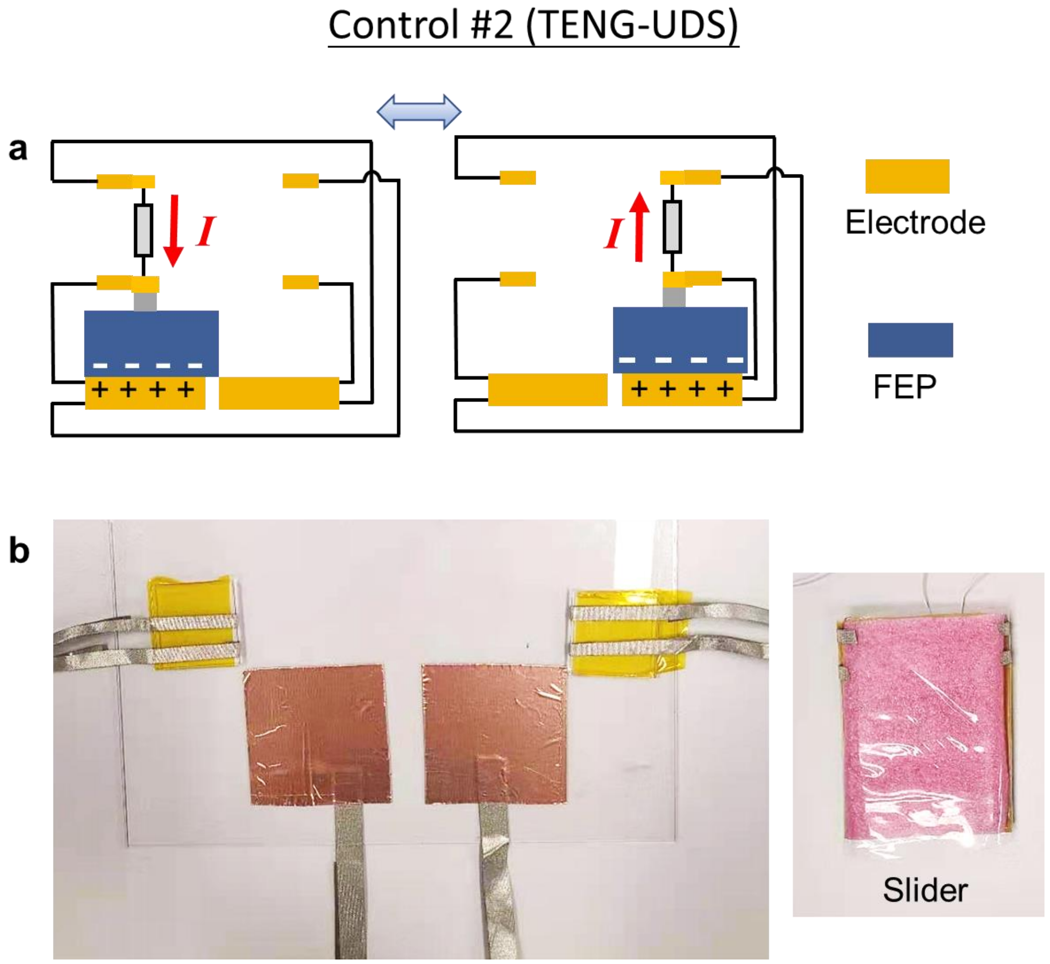

Supplementary Figure 3. (a) Schematic of the working principle of the TENG with unidirectional switch (TENG-UDS). **(b)** Photograph of TENG-UDS (Control #2). Detailed information on this device is described in Ref. [1]

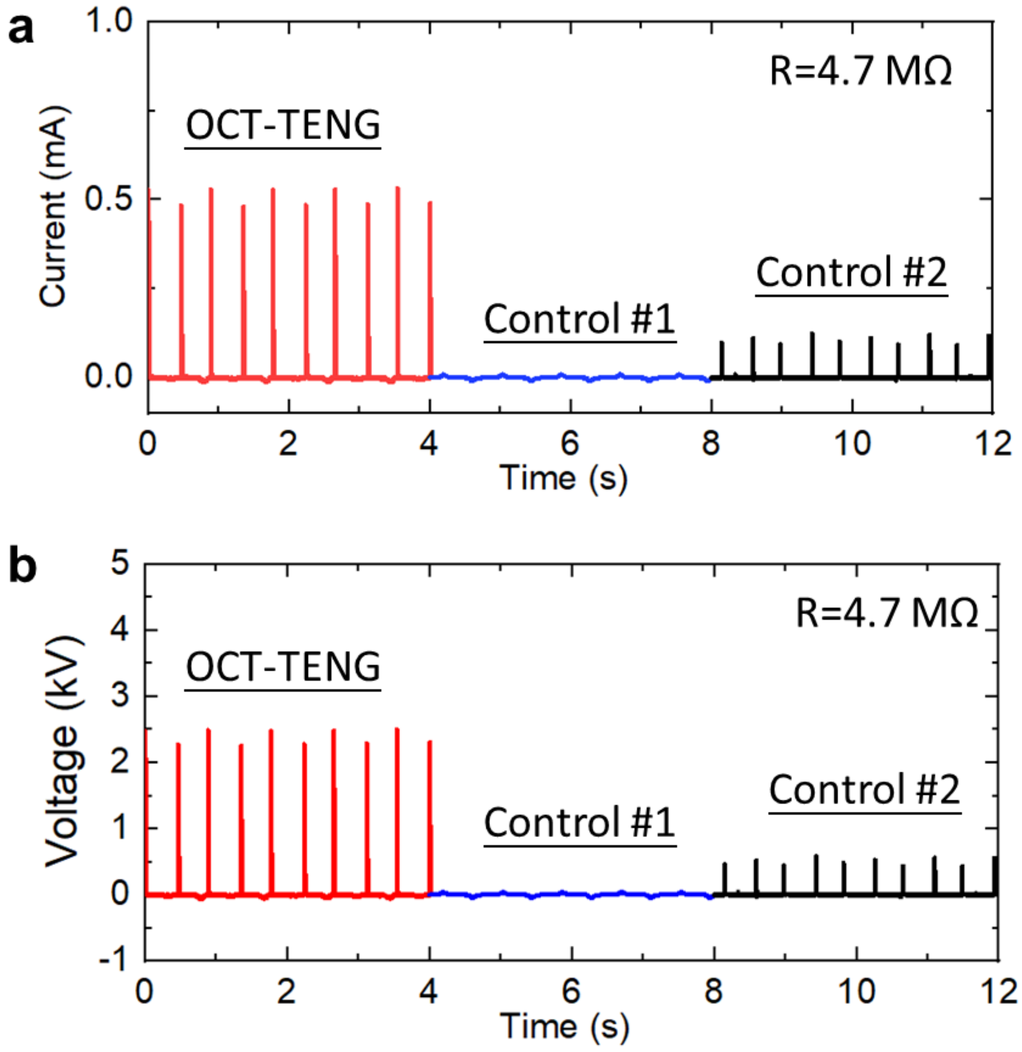

Supplementary Figure 4. (a) Current and **(b)** voltage output of the OCT-TENG, SFT-TENG (Control #1), and TENG-UDS (Control #2)

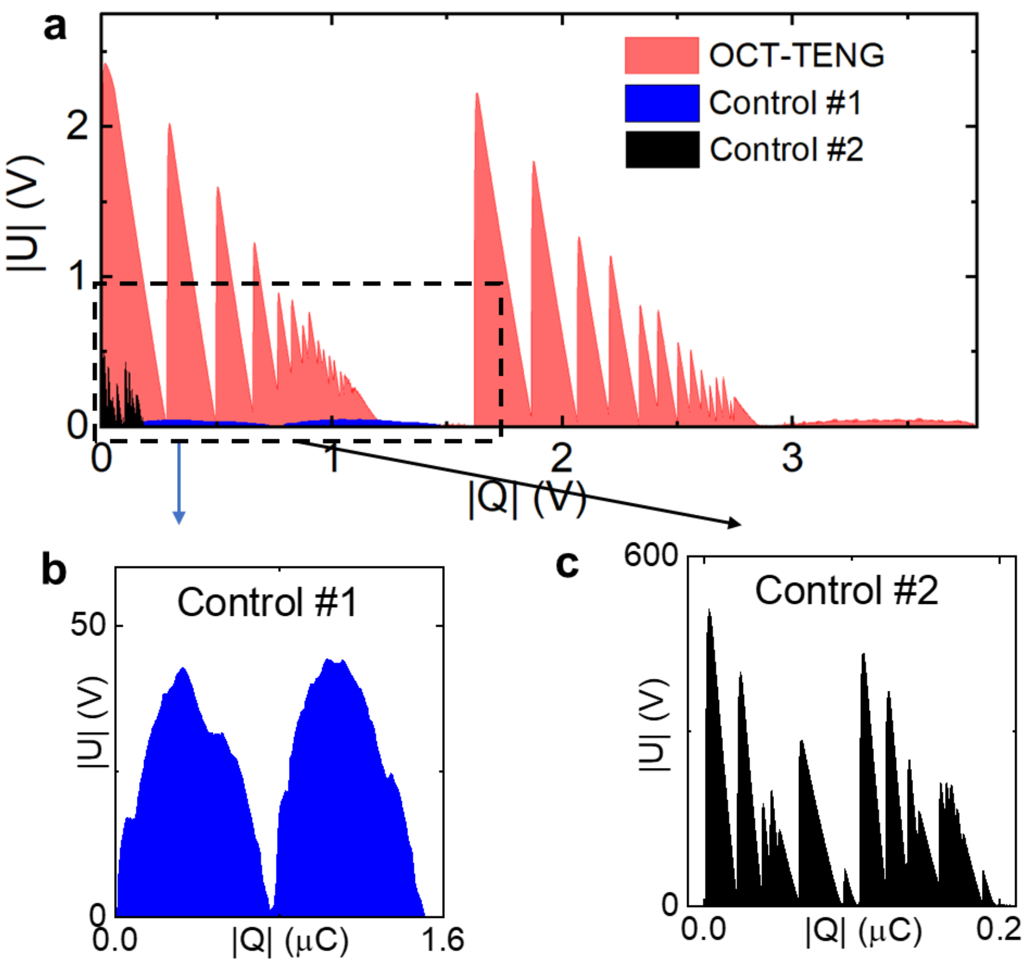

Supplementary Figure 5. U-Q plot of the **(a)** OCT-TENG, **(b)** Control #1 (SFT-TENG), and **(c)** Control #2 (TENG-UDS).

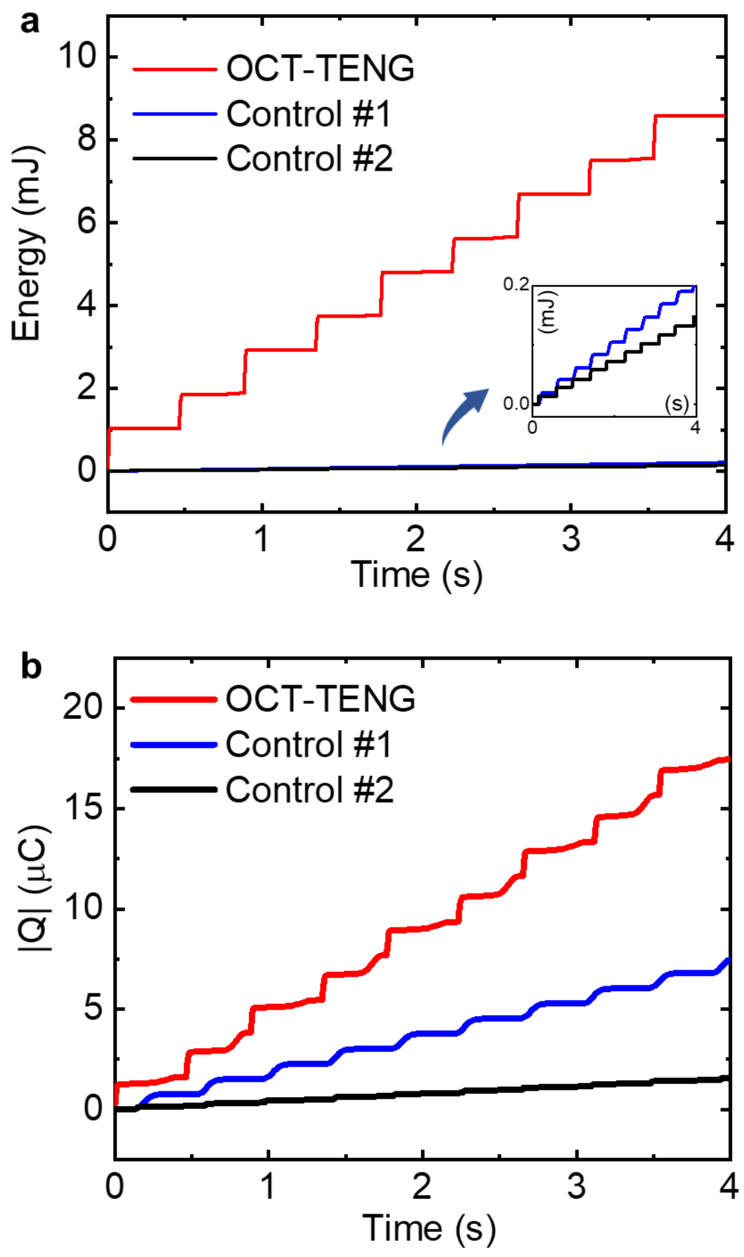

Supplementary Figure 6. Comparison of the **(a)** generated energy and **(b)** transferred charges of OCT-TENG, Control #1, and Control #2.

II. The current generation (load resistance of 22 Ω) at Stage 1 (Supplementary Figure 7)

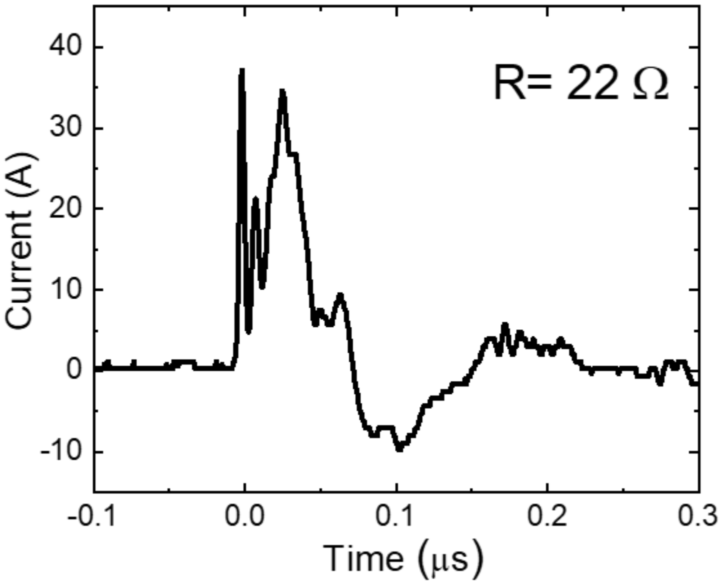

Supplementary Figure 7. Current generated from an OCT-TENG with a load resistance of 22 Ω in Stage 1.

III. Comparison of the power density of OCT-TENG compared to previous reports (Supplementary Figure 8)

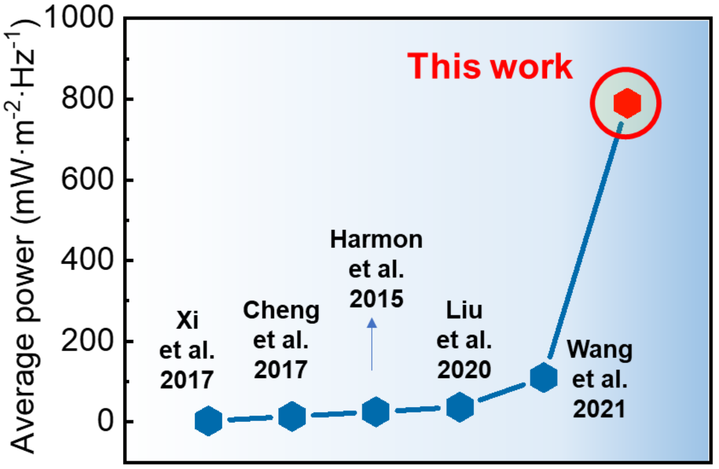

Supplementary Figure 8. The average power density per Hz of the OCT-TENG (this work) compared to the previous report.

IV. Equivalent circuit (Supplementary Figure 9)

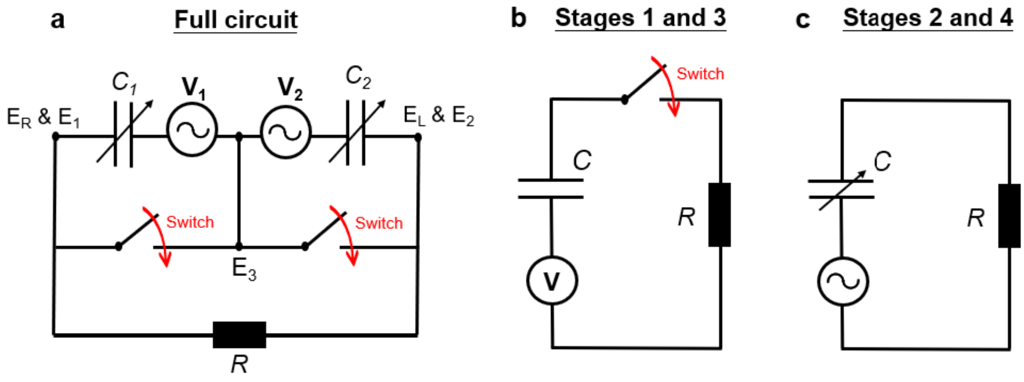

Supplementary Figure 9. **(a)** Equivalent circuit of OCT-TENG. (**b)** Equivalent circuit of OCT-TENG operated in Stage 1 and 3 (ON state). (**c)** Equivalent circuit of OCT-TENG operated in Stage 2 and 4 (OFF state).

V. Details of charge and current output of OCT-TENG (Supplementary Figure 10)

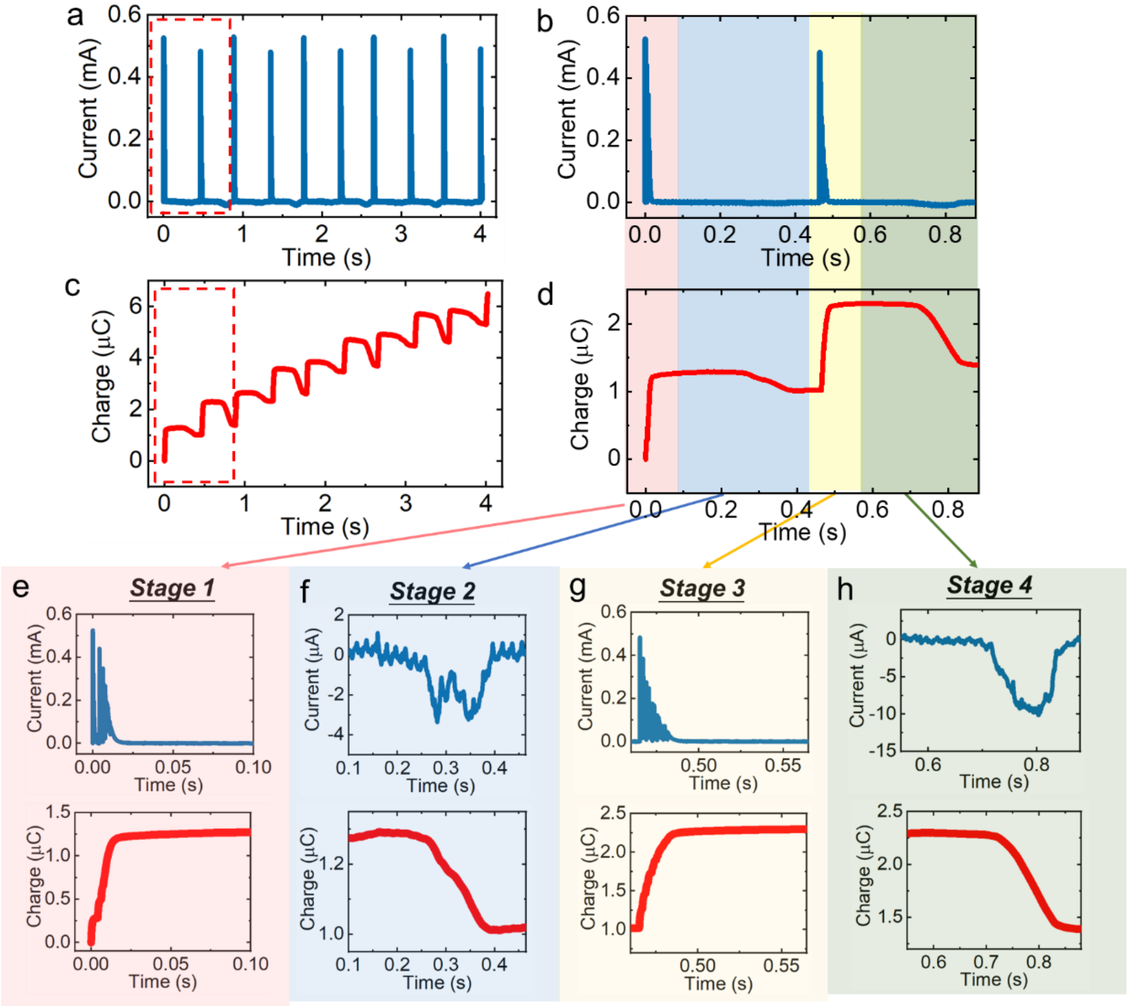

Supplementary Figure 10. **(a)** The current and **(c)** charge output of the OCT-TENG during multiple cycles of operation. **(b)** The current and **(d)** charge output of the OCT-TENG during one cycle of operation. **(e-h)** The current and charge output of each stage.

VI. Charging capacitor using OCT-TENG and Control TENG (Supplementary Figures 11-13)

We first fabricated an OCT-TENG and a control TENG, and measured their current outputs to make sure they both work well, as shown in **Supplementary Figure 11.** The operation frequency of the OCT-TENG and the control TENG are both 1.2 Hz.

Then, we tested the charging performance of the OCT-TENG and the control-TENG. The capacitor charging circuits are shown in **Supplementary Figure 12**. In both circuits of OCT-TENG and the control-TENG, a bridge rectifier is used to turn the AC into DC for the capacitor charging.

Supplementary Figure 13 shows the results of charging capacitors using the OCT-TENG and the control-TENG. With an OCT-TENG, a 10 μF capacitor can be charged to 15V within 30 s. With 80 s charging time, the capacitors of 47μF, 100 μF, and 220 μF can be charged to 10.30V, 4.15V, and 2.19 V, respectively (**Supplementary Figure 13a**). From the charging curves, we can observe the charge transfer at each stage, as shown in **Supplementary Figure 13b.** The charging performances of the control TENG with capacitors of 10μF, 47μF, 100 μF, and 220 μF are shown in **Supplementary Figure 13c** and **d**. The capacitor charging speed of the OCT-TENG is 2.7~3.0 times faster than the control TENG, as shown in **Supplementary Figure 13e-g**, conforming the opposite-charge enhancement effect of the OCT-TENG.

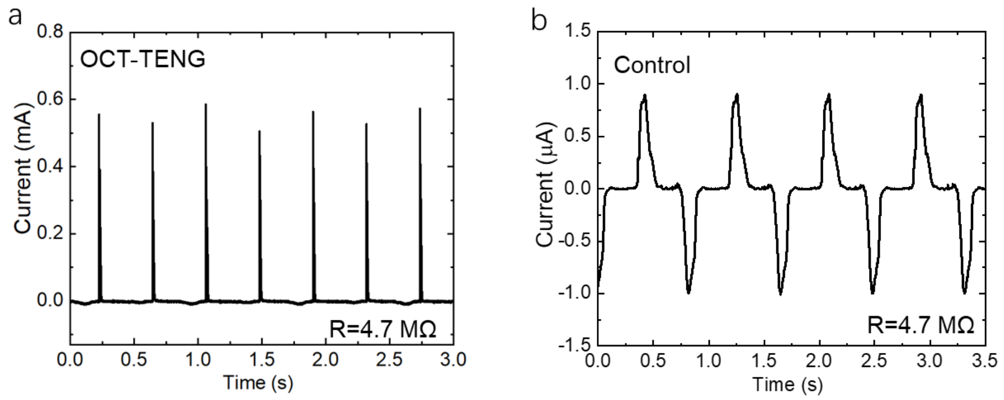

Supplementary Figure 11. The generated current of the **(a)** OCT-TENG and **(b)** control TENG with a load resistance of 4.7 MΩ. The operation frequency of the OCT-TENG and control TENG are both 1.2 Hz.

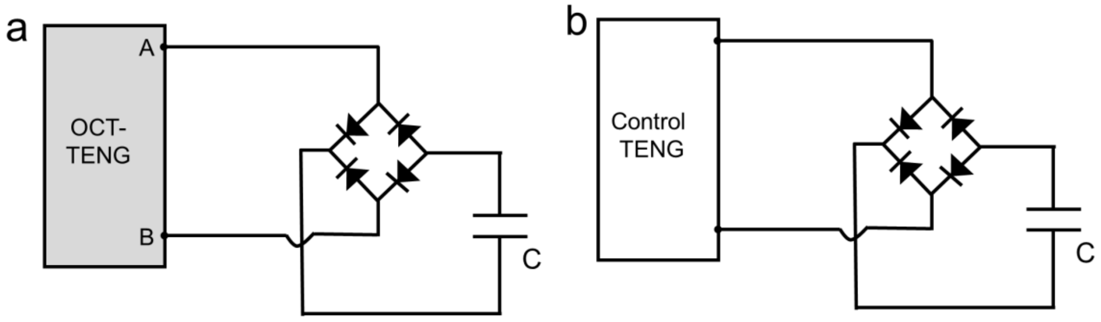

Supplementary Figure 12. The circuit of the charging the capacitors with **a,** OCT-TENG and **b,** control TENG.

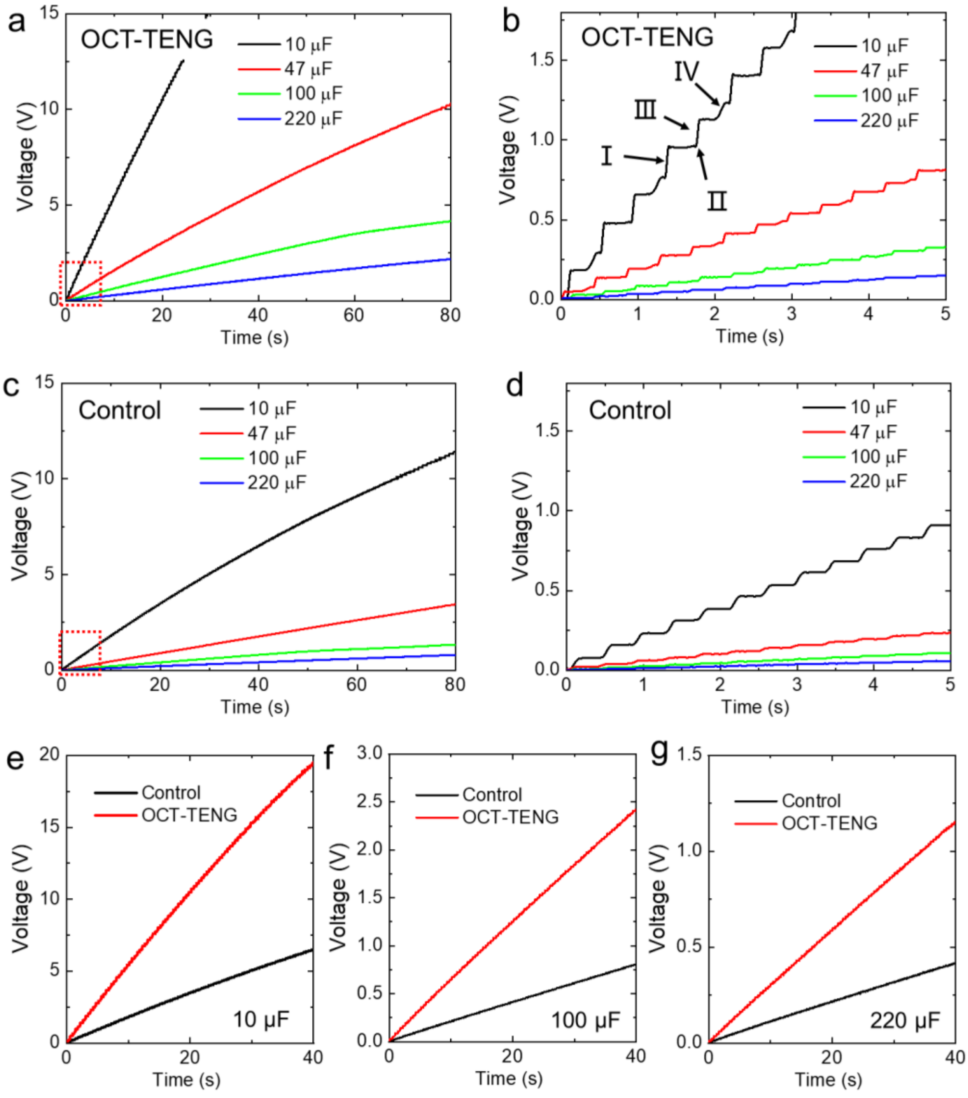

Supplementary Figure 13. The charging curves of 10 μF (black), 47 μF (red), 100 μF (green), and 220 μF (blue) capacitors with **(a, b)** OCT-TENG and **(c, d)** control TENG. The operation frequency of OCT-TENG and control TENG are both 1.2 Hz (shown in **Supplementary Figure 11**). The charging curves of **(e)** 10 μF, **(f)** 10 μF, and **(g)** 10 μF capacitors with OCT-TENG and the control TENG.

VII. Peak current depending on load resistance (Supplementary Figure14)

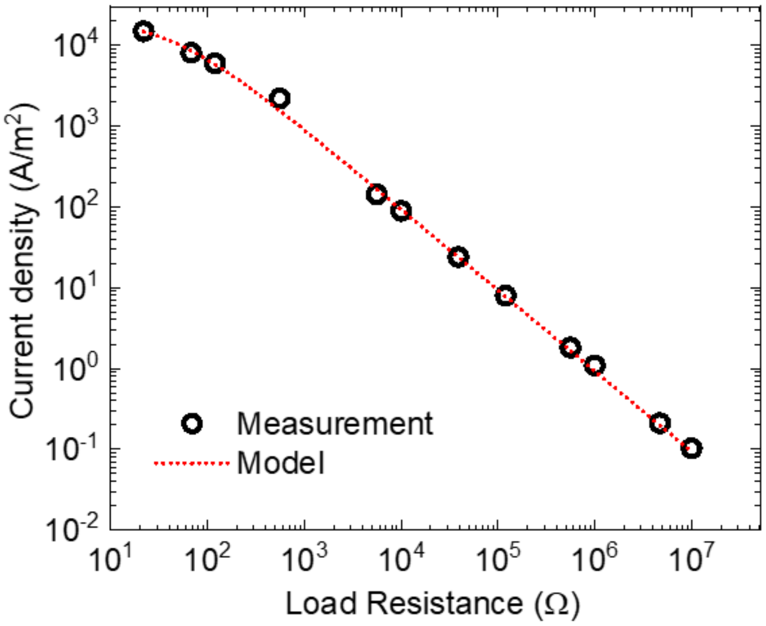

Supplementary Figure 14. The measured peak current density and the modeled value with fitted inner resistance of 41 Ω.

VIII. Photograph of operation of OCT-TENG (Supplementary Figure15)

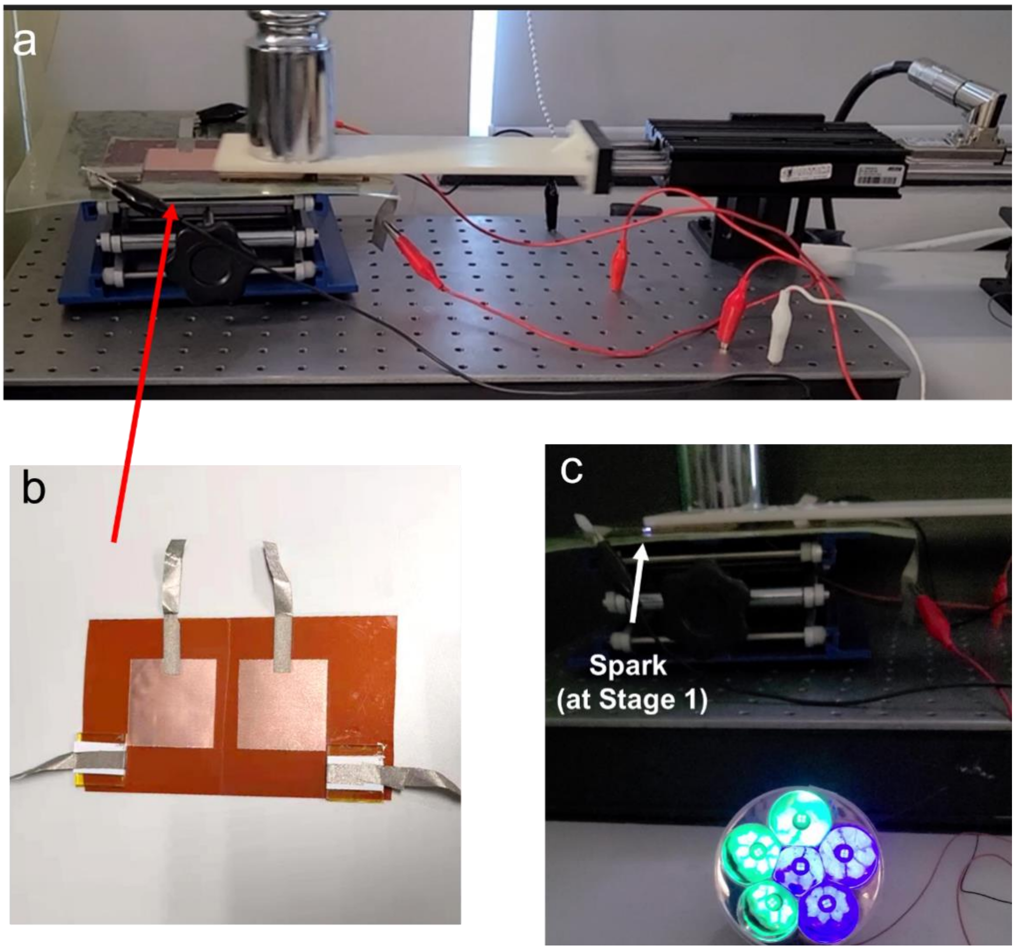

Supplementary Figure 15. (a) The photograph of the OCT-TENG operated by a linear motor. **(b)** The photograph of the OCT-TENG. **c.** Photograph showing the spark when the electrode on the slider touches the floating electrode E_L.

IX. The U-Q plot of the OCT-TENG (varying load resistance) (Supplementary Figure 16)

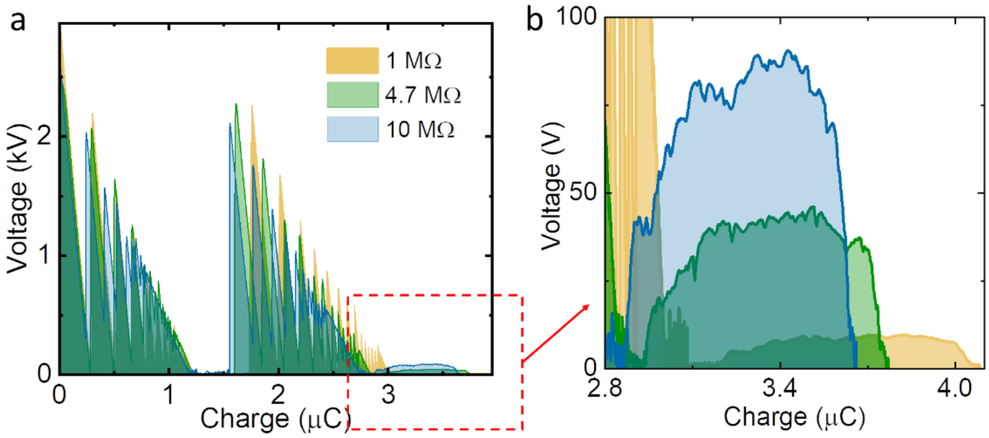

Supplementary Figure 16. (a) The U-Q plot of the OCT-TENG with the load resistance. **(b)** The zoomed-in U-Q plot of OCT-TENG in Stage 4.

X. Experiments based on varying parameters (including the effective area, the distance between E_L and E_R, and the shape of the electrode on the stator) (Supplementary Figures 17-20)

(1) Varying the effective area of the OCT-TENG

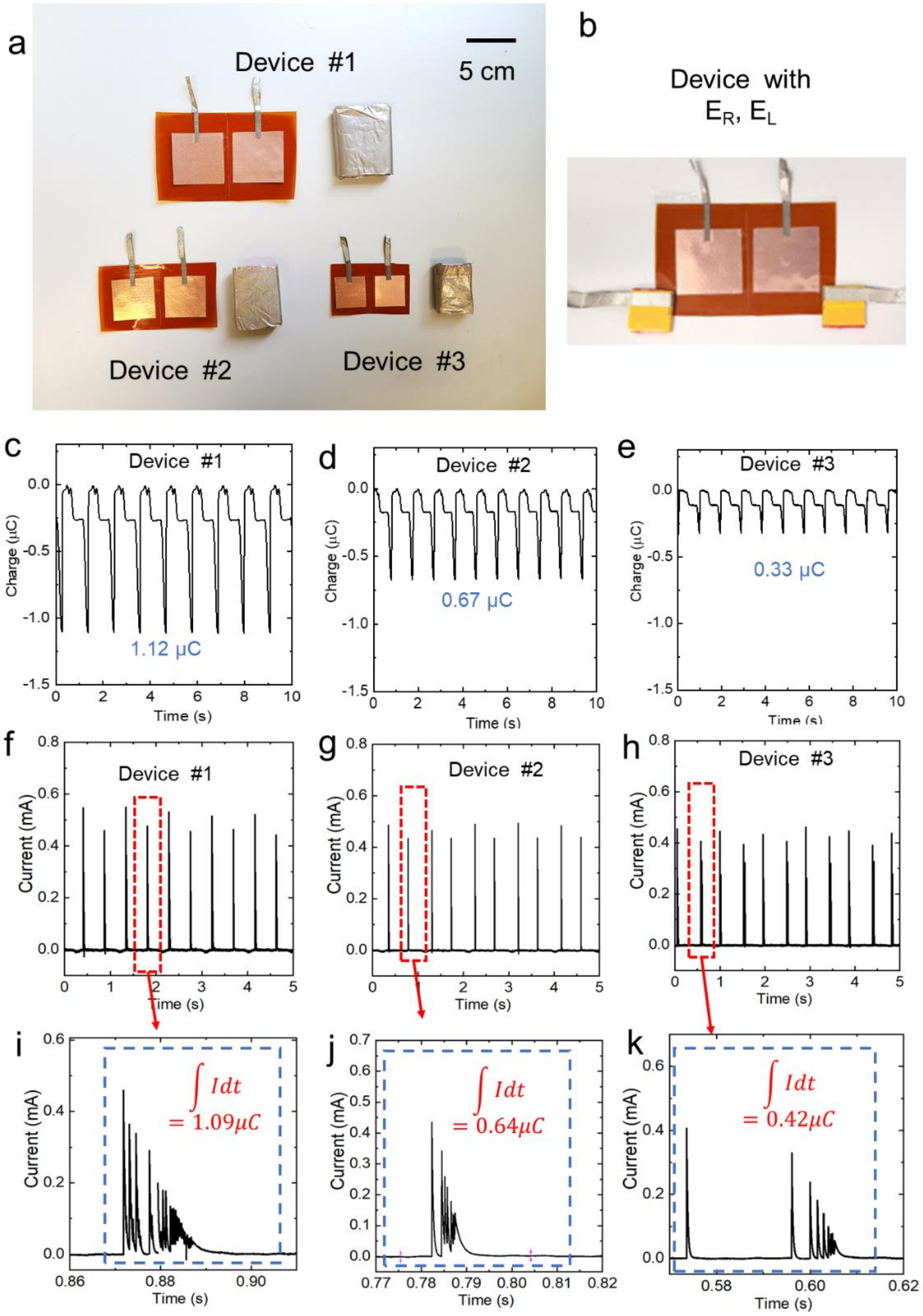

Supplementary Figure 17. (a) Photograph of the OCT-TENGs (without "Gate" electrodes of E_L and E_R) with virous effective area of 5 cm × 5 cm (Device #1), 4 cm ×

4 cm (Device #2), and 3 cm × 3 cm (Device #3). **(b)** Photograph of the OCT-TENG with electrodes of E_L and E_R. Charge output of **(c)** Device #1, **(d)** Device #2, and **(e)** Device #3 detected via the "four-ports" methodology. Current outputs of **(f)** Device #1, **(g)** Device #2, and **(h)** Device #3 with the load resistance of 4.7 MΩ. The generated current of the OCT-TENG at the "ON" state of **(i)** Device #1, **(j)** Device #2, and **(k)** Device #3 with the load resistance of 4.7 MΩ. The values of $|Q_2 - Q_1|$ of Device #1, Device #2, and Device #3 calculated from the current integration are shown in (**i** – **k**) in red.

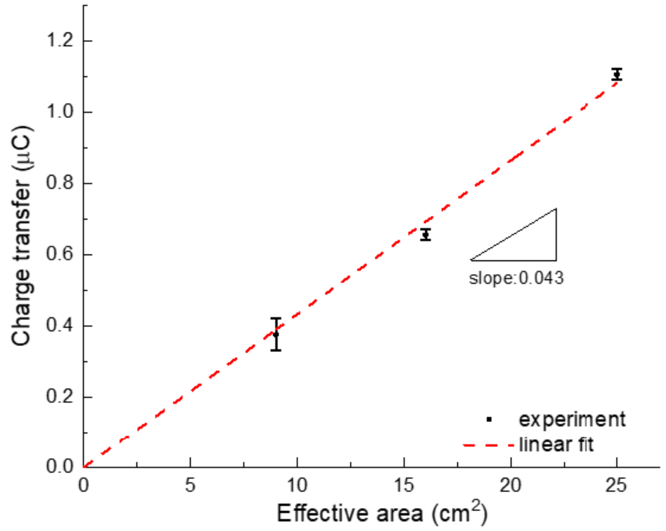

Supplementary Figure 18. The charge transfer at ON state (stage 1 or 3) depending on effective area of OCT-TENG. The error bars are from the measured data via "four-"four-ports" methodology and the standard measurement.

In theory, according to our model, with the identical material system the charge output should be proportional to the area of the effective area of the sample. Because the charge transfer at the "ON" stage is $Q_2 - Q_1 = (\sigma_2 - \sigma_1)A$, where A is the effective area (referred to one Cu electrode area on the stator in this work), the σ_1 and σ_2 are the charge densities corresponding to $Q_1$and Q_2. With the identical material system, the generated charge densities on the material's surface should be roughly the same.

We performed experiments on the devices with reduced effective area, and the results is shown in **Supplementary Figure 17**. **Supplementary Figure 17a** demonstrated the photos of the three OCT-TENG devices fabricated with effective areas of 5 cm × 5 cm (Device #1), 4 cm × 4 cm (Device #2), and 3 cm × 3 cm (Device #3). In this comparison experiment, we use the same electrode of E_L and E_R for the Device #1, Device #2 and Device #3. The OCT-TENG with E_L and E_R is shown in **Supplementary Figure 17b**.

We first tested the charge output using the "four-ports" methodology which has been demonstrated in Fig. 4 in our manuscript. As shown in **Supplementary Figure 17c-e,** $Q_2 - Q_1$ of Device #1, Device #2, and Device #3 detected using the "four-ports" methodology are 1.12 μC, 0.67 μC, and 0.33 μC respectively. It is clear that the charge output of $Q_2 - Q_1$ is scaled with the effective area.

We then compared the current outputs (load resistance: 4.7 MΩ), and the results are shown in **Supplementary Figure 17f-k**. The current of the three devices are roughly similar, and the peak values are slightly decreased with the area decreasing. At the "ON" stage, charges of $Q_2 - Q_1 = (\sigma_2 - \sigma_1)A$ immediately transfer between the "Source" and the "Drain". The current peak $I_P = \frac{U}{R} \propto \frac{(\sigma_2 - \sigma_1)}{cR}$, where the $(\sigma_2 - \sigma_1)$ are the unbalanced charges, and c is the dielectric capacitance per area, the R is the load resistance. As a result, the current peak value is in theory identical when varying the area due to the similar $\sigma_2 - \sigma_1$. However, practically, when the area is small, the total transferred charges at the "ON" stage ($Q_2 - Q_1$) is relatively small and is easier to be affected by factors such as the testing instruments, the circuit, and the environment.

Despite the similar peak currents, the charge transfer at the "ON" stages integrated from the current show that the $Q_2 - Q_1$ of the three devices decrease with the active area decreasing. The value of $Q_2 - Q_1$ integrated from the current at the "ON" state are 1.09 μC, 0.64 μC and 0.42 μC for Device #1, Device #2 and Device #3, respectively. These values are consistent with the results tested using the "four-ports" methodology. Thus, the charge output decreases linearly with the size of the device. (**Supplementary**

Figure 18)

(2) Varying the distance of E_L and E_R

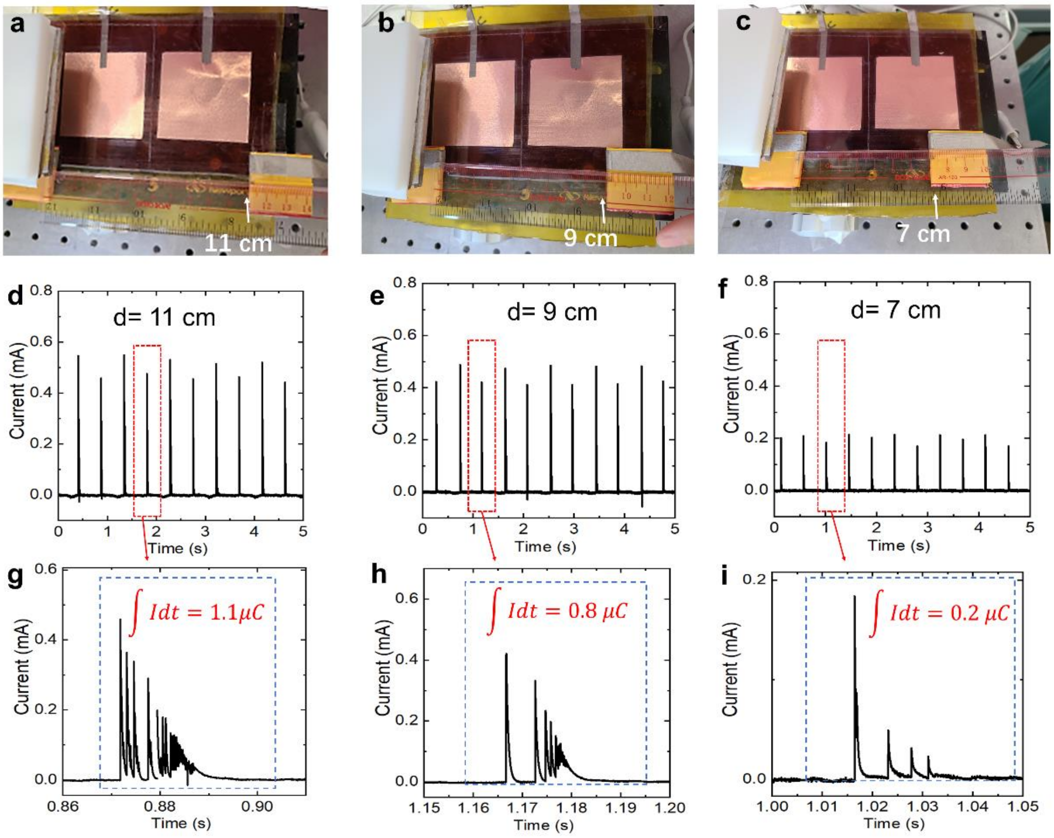

Supplementary Figure 19. The photographs of the OCT-TENG (effective area of 25 cm^2) with the distance of E_L and E_R of **(a)** 11 cm, **(b)** 9 cm, and **(c)** 7 cm. The generated current of the OCT-TENG with the distance of E_L and E_L of **(d)** 11 cm**, (e)** 9 cm, and **(f)** 7 cm. (load resistance is 4.7 MΩ). The charge transfer, $|Q_2 - Q_1|$, of the OCT-TENG at the "ON" state calculated from the current integration with the distance of E_L and E_R of **(g)** 11 cm, **(h)** 9 cm, and **(i)** 7 cm. (load resistance is 4.7 MΩ).

As shown in **Supplementary Figure 19 a-c**, we set the distance d of the E_L and E_R as 11 cm, 9 cm, and 7 cm, respectively. **Supplementary Figure 19 d-e** show the generated current when varying the d. Although the area of the device is kept constant, the effective area is decreased due to the decrease of d. As a result, the transferred

charge $Q_2 - Q_1$ at the "ON" stage decreased with the decrease of the d. As shown in **Supplementary Figure 19 d-i**.

(3) Varying the shape of the electrodes on the stator

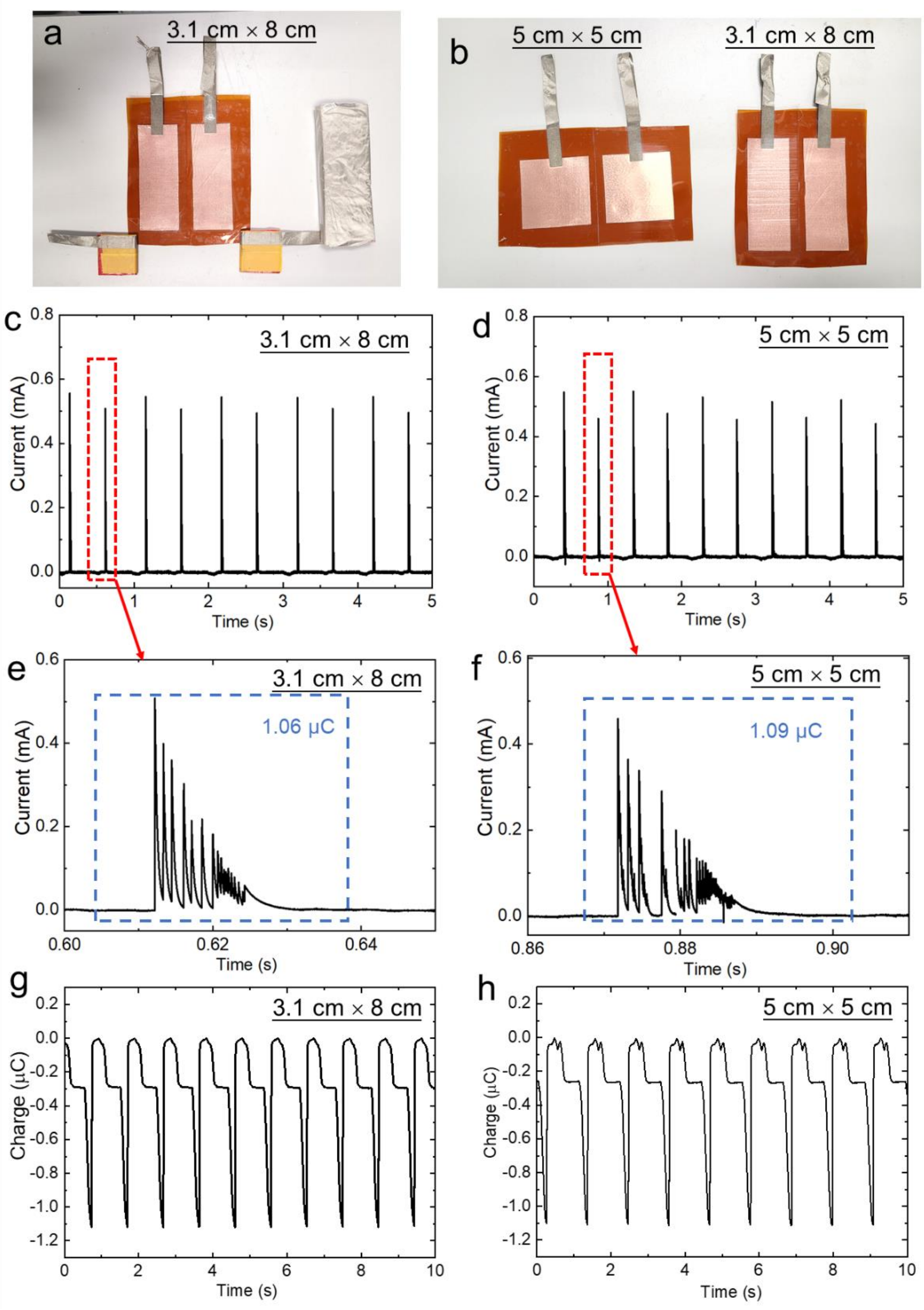

Supplementary Figure 20. (a) Photograph of the OCT-TENG with effective area of 3.1 cm × 8 cm ≈ 25 cm^2. **(b)** Photograph of the stators of the OCT-TENG with the

effective area of 3.1 cm × 8 cm and 5 cm × 5 cm. The generated current of the OCT-TENG with area of **(c)** 3.1 cm × 8 cm and **(d)** 5 cm × 5 cm. The charge transfer, $|Q_2 - Q_1|$, at the "ON" state calculated from the current integration of OCT-TENG with area of **(e)** 3.1 cm × 8 cm and **(f)** 5 cm × 5 cm. Charge output of OCT-TNEG with area of **(g)** 3.1 cm × 8 cm and **(h)** 5 cm × 5 cm.

We performed experiments and compared the outputs of two OCT-TENG with identical effective area but different electrode shapes, also corresponding to different distance between E_L and E_R. The photograph of OCT-TENG with effective area of 3.1 cm × 8 cm ≈ 25 cm^2 is shown in **Supplementary Figure 20a**. As a comparison, the standard OCT-TENG with area of 5 cm × 5 cm = 25 cm^2 is shown in **Supplementary Figure 20b**. As shown in Fig. **Supplementary Figure 20c-d**, the current outputs (load resistance of 4.7 MΩ) of these two devices are very similar. The $Q_2 - Q_1$ calculated from the integral of the current at "ON" state are also similar, as shown in **Supplementary Figure 20e-f**. We also performed the "four-ports" methodology to further detect the quantity of the charge transfer, and we found the measured $Q_2 - Q_1$ for these two devices are both 1.12 μC, as shown in **Supplementary Figure 20 g-h.** So, although the shapes of these two devices are different, as long as their effective area are identical the outputs will be identical.

XI. Schematic of the "four-ports" method and experimental results (Supplementary Figures 21-24)

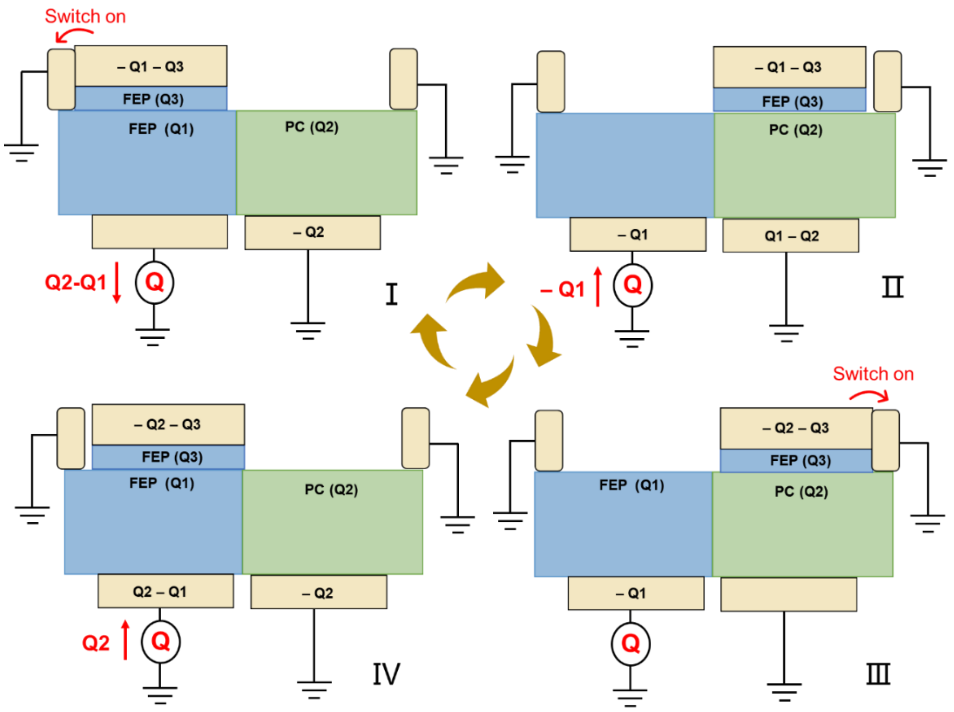

Supplementary Figure 21. The schematic of the charge output from Electrode E_1 and when the electrodes E_1, E_2, E_L, and E_R are grounded.

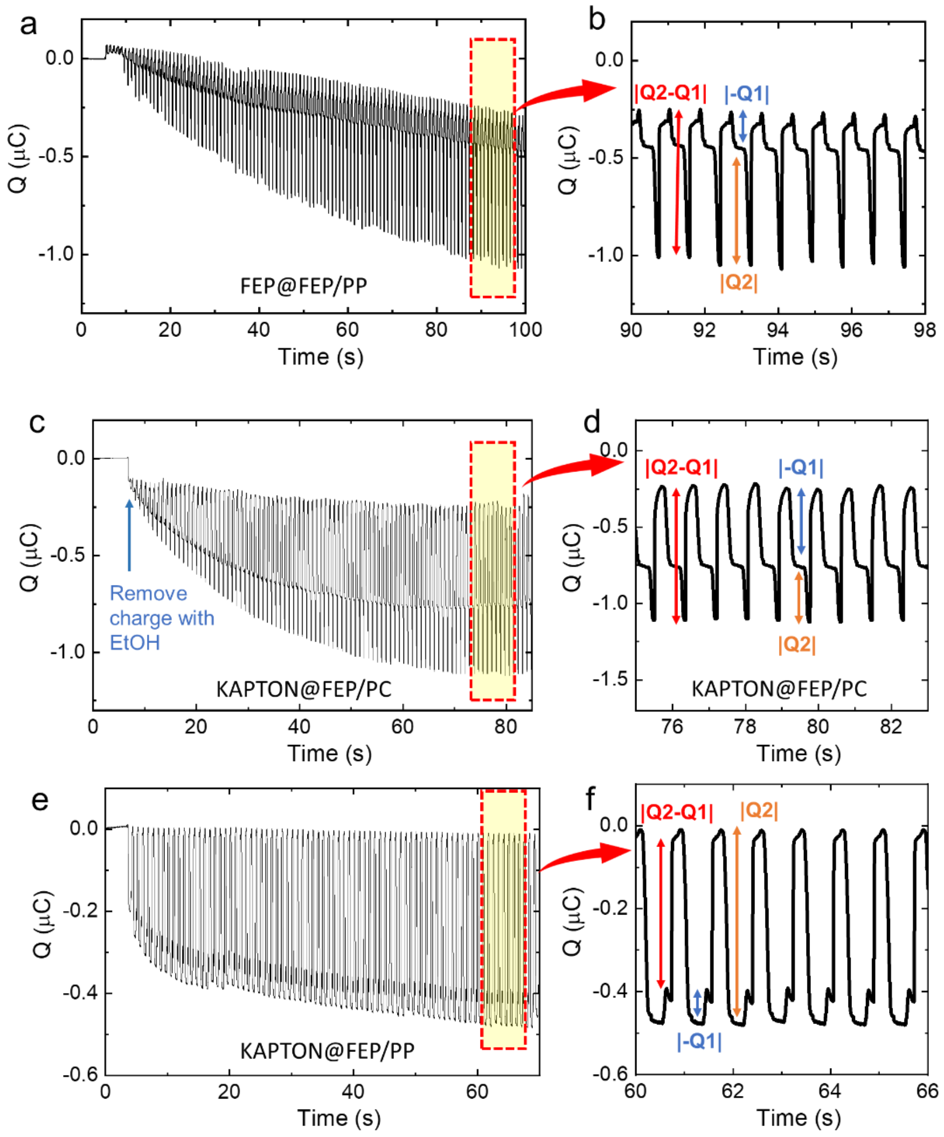

Supplementary Figure 22. Charge accumulation detected using "four-ports" method on the samples of **(a)** FEP@FEP/PP, **(c)** Kapton @FEP/PC and **(e)** Kapton @FEP/PP. The measured $Q1$ and $Q2$ from samples of **(b)** FEP@FEP/PP, **(d)** Kapton @FEP/PC and **(f)** Kapton @FEP/PP.

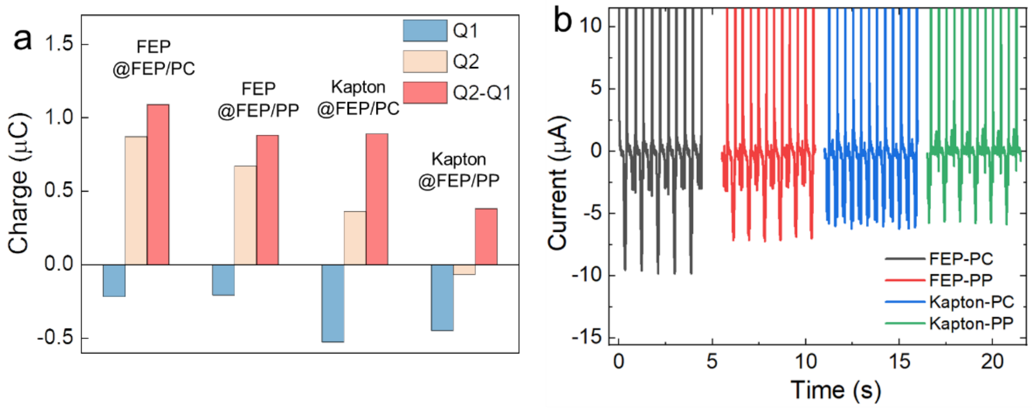

Supplementary Figure 23. **(a)** Measured Q_1, Q_2 and $Q_2 - Q_1$ with various material systems. (A@B/C refers to the tribo-surface of the slider as A and that of the stator as B and C) **(b)**The negative current peaks generated from OCT-TENGs with various tribo-material pairs.

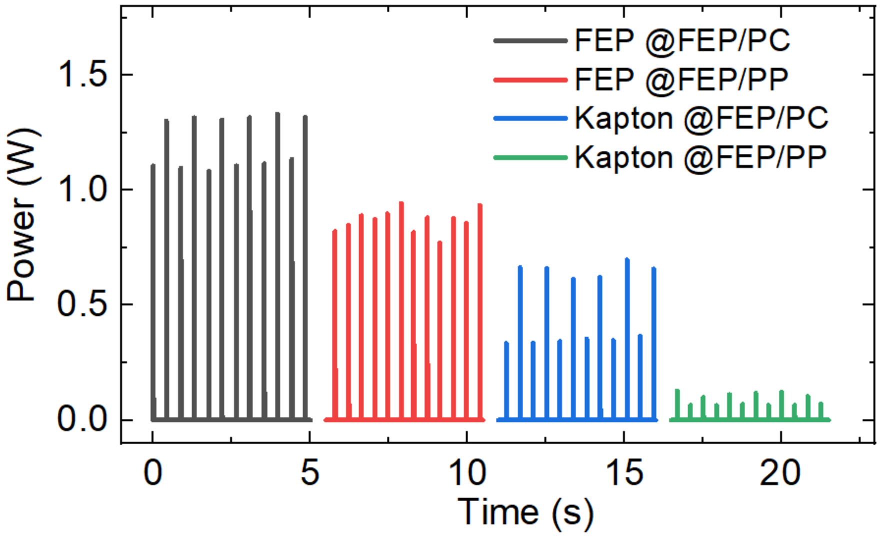

Supplementary Figure 24. The generated power from OCT-TENG of samples with various material systems, including FEP@FEP/PC, FEP@FEP/PP, Kapton@ FEP/PC and Kapton@ FEP/PP.

XII. Tribo-charges generation on ternary materials (Supplementary Figure 25)

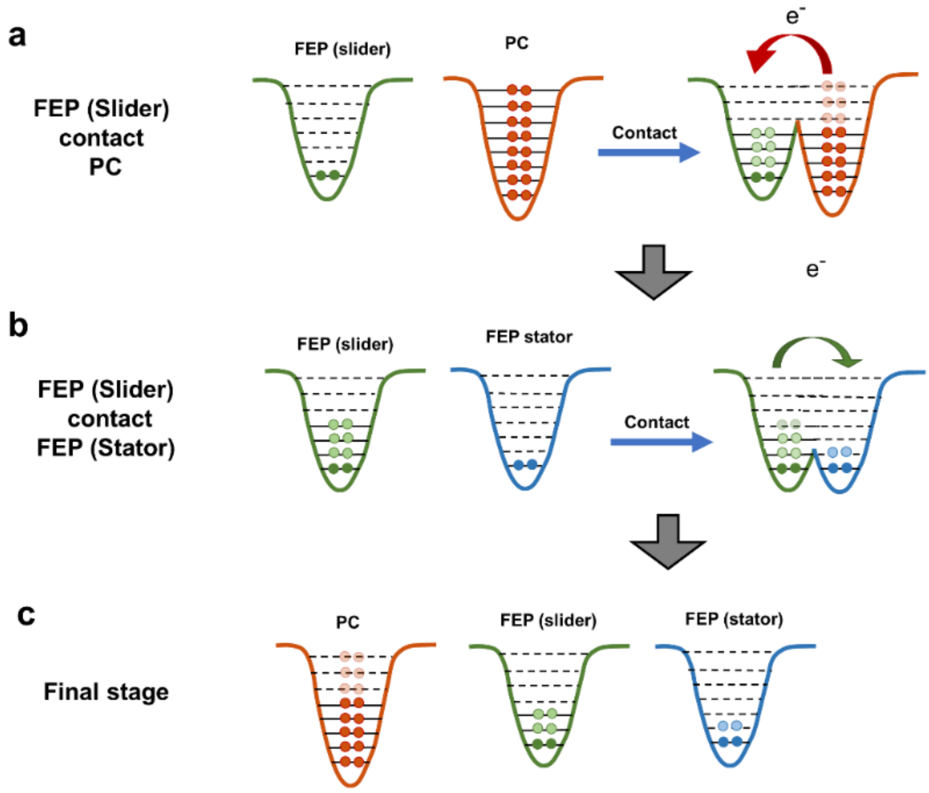

Supplementary Figure 25. Explanation of the FEP surface on the stator gets negatively charged after contacting with FEP on the slide by electron cloud-potential well model. **(a)** Electrons transfer from the PC surface to the FEP surface on the slider when the FEP on the slider contacts the PC surface **(b)** Electrons transfer from the FEP surface on the slider to the FEP surface on the stator when the FEP on the slider contacts the FEP surface on the stator. **(c)** After the contact of the three surfaces, positive charges are generated on the PC surface, and negative charges are generated on the FEP surfaces on both slider and the stator.

XIII. The stability and durability of OCT-TENG (Supplementary Figure 26-28)

We did not apply the "high voltage polarization process" in the fabrication of the OCT-TENG. So, there is no charge decay problem in our device. We re-tested our sample (in Jun. 2021) which was fabricated 6 months ago (in Dec. 2020), and the output does not change after 6 months stored in an ambient environment in our lab, as shown in **Supplementary Figure 26**

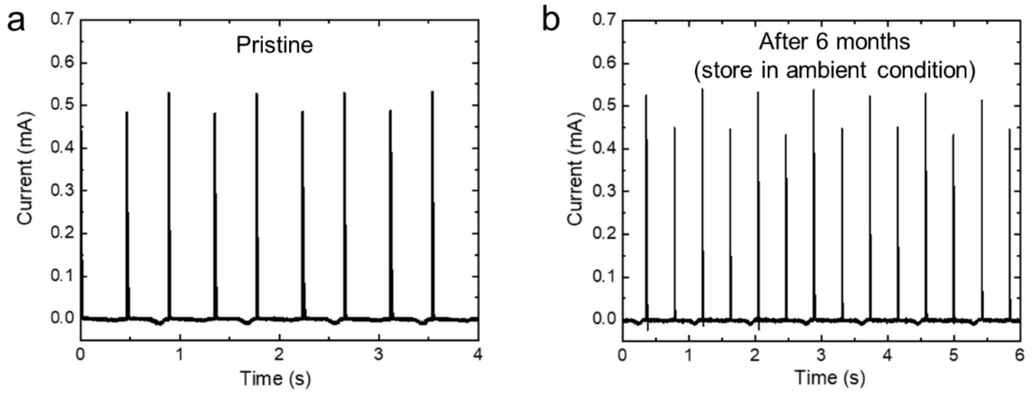

Supplementary Figure 26. Current outputs of the pristine OCT-TENG and the OCT-TENG stored in room environment for 6 months (load resistance: 4.7 MΩ).

We also performed a 10,000 cycles durability test with the OCT-TENG. Similar to all the TENGs that operated in a contact mode, the surface wear is the main issue for the OCT-TENG. Photograph of the samples before and after aging test has been shown in **Supplementary Figure 27a** and **b**. We also compared the virgin and aged materials' surfaces under microscope, as shown in **Supplementary Figure 27d**. For the optical microscope observation, to avoid the influence of the electrode background (such as Cu), we first peel the tribo-layers from the substrate and then observe it under microscope. The pealing process is shown in **Supplementary Figure 27c**. As shown in **Supplementary Figure 27d**, we found the wear on the PC surface was not obvious, while it's clear that the FEP surfaces have been worn. Considering that FEP is one of the most popular tribo-materials that is widely used in the TENG devices, addressing its wearing issue is an interesting topic. However, it's out of the scope of the current study.

The results of the electric output during the reliability test are shown in **Supplementary Figure 28**. Within 2500 cycles, there is no degradation of the output of the OCT-TENG. Current degradation has been observed from 3700 cycles. After 10000 cycles, the current and voltage outputs show ~ 20 % degradation. This was mainly caused by the wearing of the 10 μm thin FEP film on the slider. From the surface observation of the microscope shown in **Supplementary Figure 28d** we can also observe that the FEP surface on the slider was obviously worn after aging. The electric output can fully recover after simply replacing the worn FEP film with a new FEP film on the slider, as shown in **Supplementary Figure 28h-j**. Hence, even if there is a need for maintenance after long-term operation, it will be very quick and convenient.

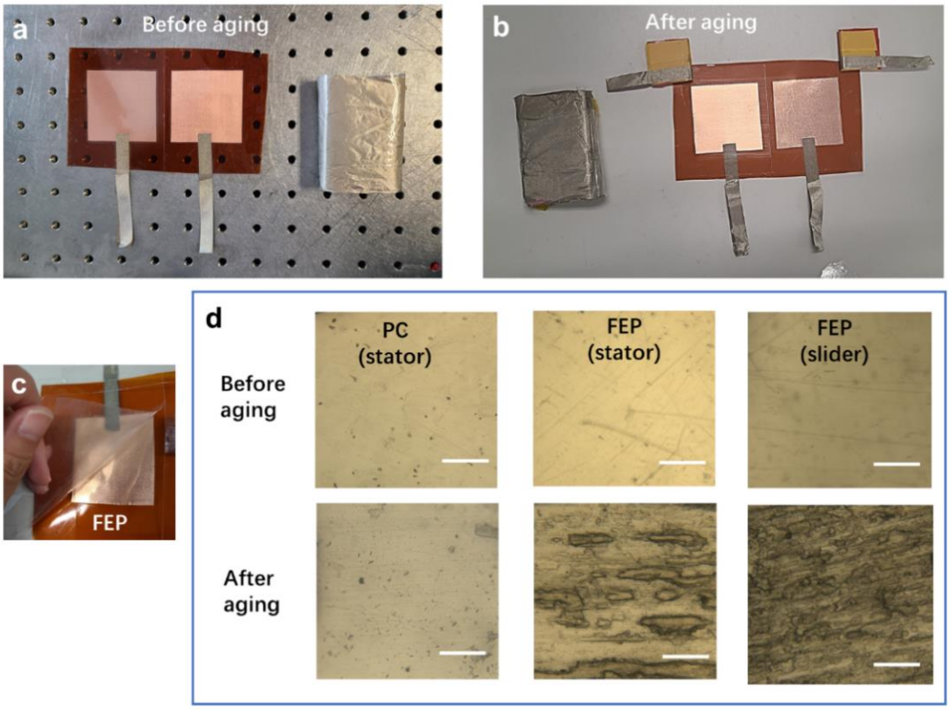

Supplementary Figure 27. Photographs of the OCT-TENG **(a)** before aging and **(b)** after aging. **(c)** Photograph of peeling the tribo-layer for the optical detection. **(d)** Observation of the tribo-surfaces (PC on the stator, FEP on the stator, and FEP on the slider) via optical microscope before and after aging. Scale bar: 500 μm.

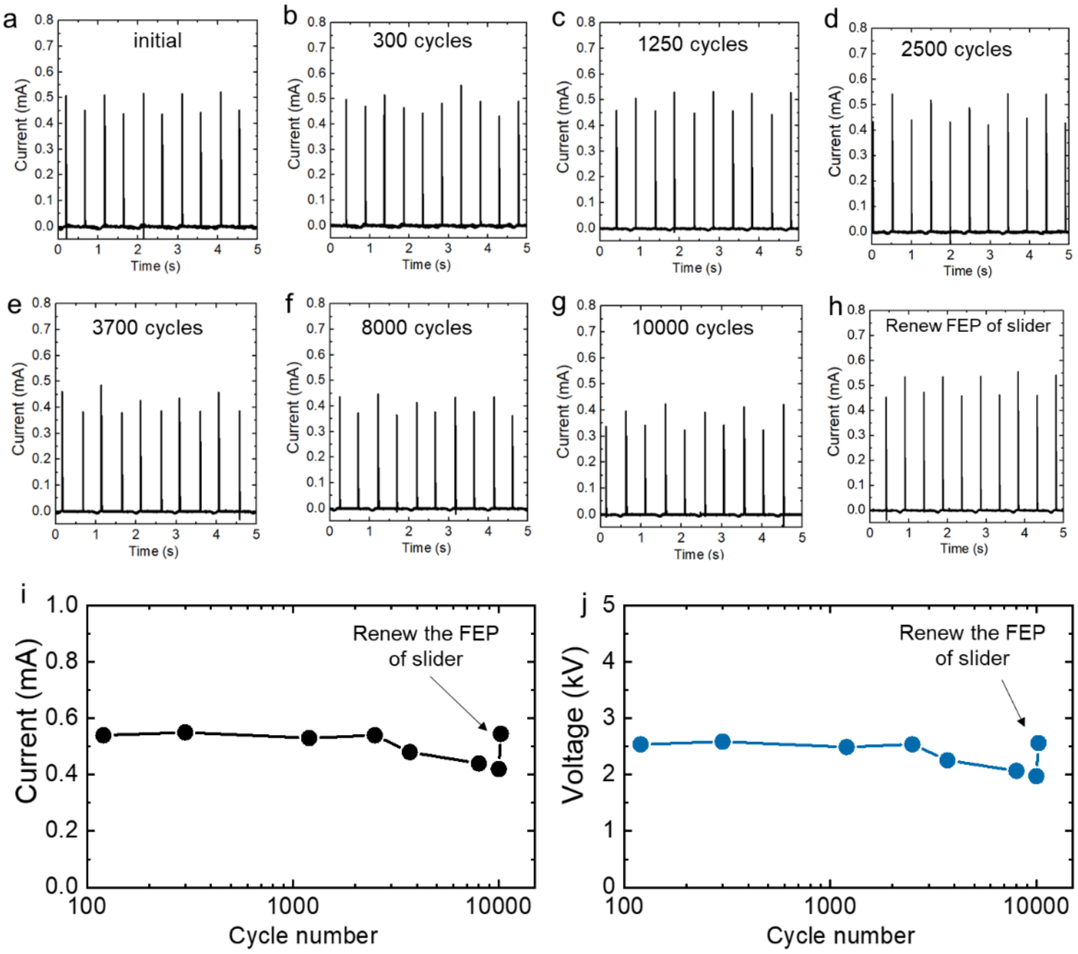

Supplementary Figure 28. (a-h) The current output of the OCT-TENG with a load resistor of 4.7 MΩ after virous cycles' operation. The operation frequency is ~ 1 Hz. **(i)** Peak current depending on the cycle number. **(j)** Peak voltage depending on the cycle number.

XIV. Powering watch and thermometer using OCT-TENG (Supplementary Figure 29)

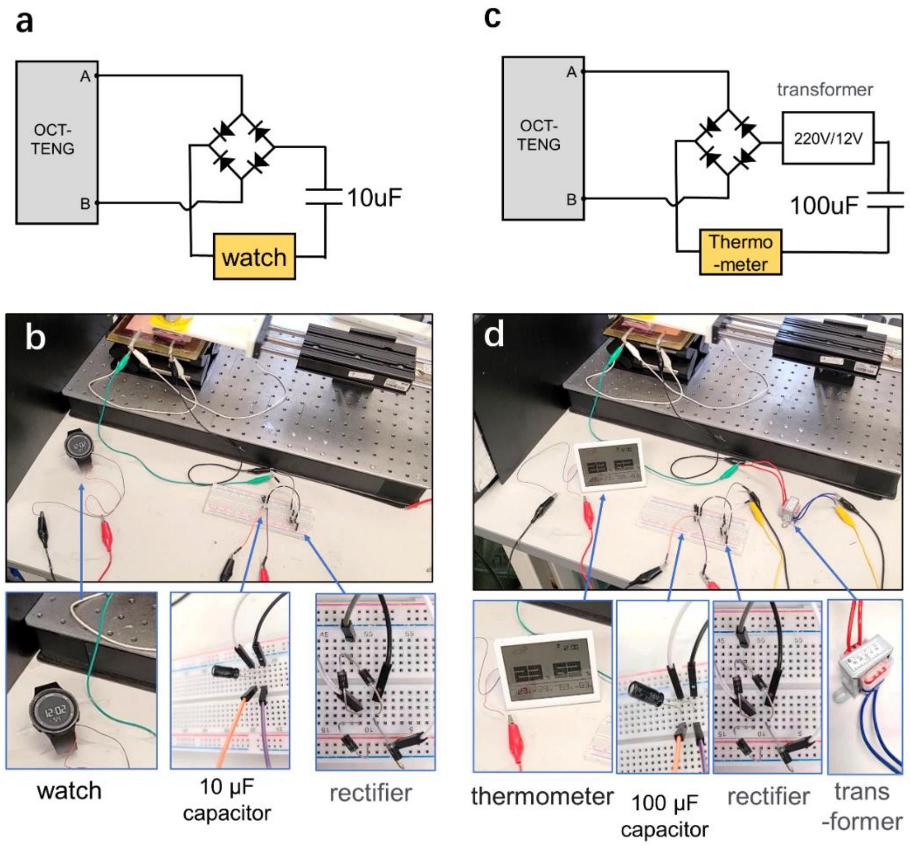

Supplementary Figure 29 (a) The circuit and **(b)** the photograph of powering a sport watch using the OCT-TENG (operation frequency: 0.8 Hz). **(c)**The circuit and **(d)** the photograph of powering a thermometer using the OCT-TENG (operation frequency: 0.8 Hz).

XV. The energy output of the OCT-TENG (direct and wireless) (Supplementary Figure 30)

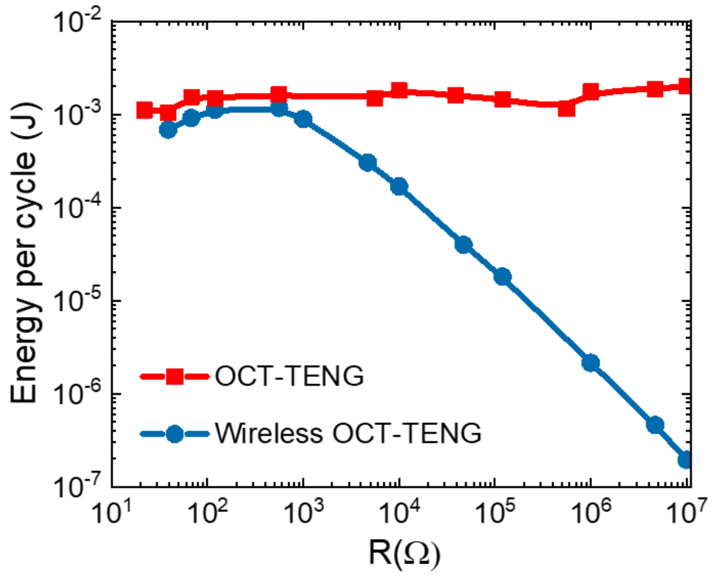

Supplementary Figure 30. The energy output of the OCT-TENG and the wirelessly delivered energy per cycle depending on the load resistance.

XVI.The current measurement circuit for the U-Q curve (Supplementary Figure 31)

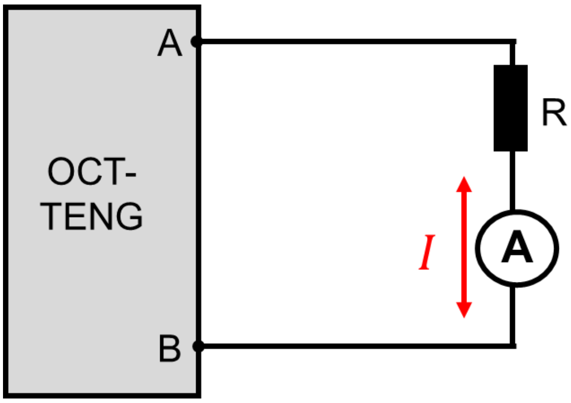

Supplementary Figure 31. The current measurement circuit for the U-Q curve.

Supplementary Note 1. Calculation of the charge transfer in OCT-TENG (Supplementary Figure 32-37)

a. Schematic and the symbol definition

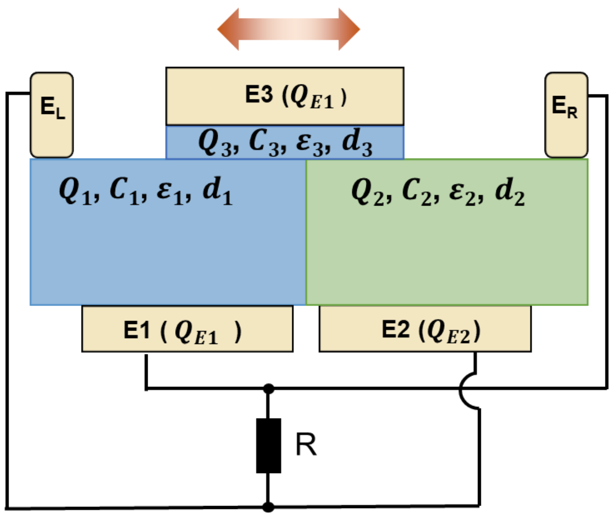

Supplementary Figure 32. The schematic of the structure of HP-TENG.

I) Symbols of materials and components:

Dielectric film #1: The dielectric material on the left side of the HP-TENG (In a typical sample, the Dielectric film #1 is FEP)

Dielectric film #2: The dielectric material on the right side of the HP-TENG (In a typical sample, the Dielectric film #2 is PC)

Dielectric film #3: The dielectric material on the right side of the HP-TENG (In a typical sample, the Dielectric film #3 is FEP on the slider)

Electrode E1: The electrode beneath Dielectric film #1

Electrode E1: The electrode beneath Dielectric film #2

Electrode E1: The electrode beneath Dielectric film #3

Electrode E_L: The electrode at the left side of the HP-TENG (labeled in the **Supplementary Figure 32.**)

Electrode E_R: The electrode at the right side of the HP-TENG (labeled in the **Supplementary Figure 32.**)

Physical Symbols:

$\boldsymbol{Q_{E1\_stage\ N}}$, $\boldsymbol{Q_{E2\_stage\ N}}$, and $\boldsymbol{Q_{E3\_stage\ N}}$: The amount of charges at the end of stage *N* in the Electrode E1, Electrode E2 and Electrode E3, respectively. (N=1, 2, 3, 4)

$\boldsymbol{Q_1}$, $\boldsymbol{Q_2}$, and $\boldsymbol{Q_3}$: The amount of Charge on the surface of Dielectric film #1, Dielectric film #2 and Dielectric film #3, respectively.

$\boldsymbol{C_1}$, $\boldsymbol{C_2}$, and $\boldsymbol{C_3}$: The capacitance of the Dielectric film #1, Dielectric film #2 and Dielectric film #3, respectively.

$\boldsymbol{\varepsilon_1}$, $\boldsymbol{\varepsilon_2}$, and $\boldsymbol{\varepsilon_3}$: The dielectric constant of Dielectric film #1, Dielectric film #2 and Dielectric film #3, respectively.

d_1, d_2 , d_3: The thicknesses of Dielectric film #1, Dielectric film #2 and Dielectric film #3, respectively.

$\boldsymbol{Q_{stage\ N\ (A\ to\ B)}}$: The amount of charge transferred from Point A to Point B (labeled in the **Fig. 2A**) at Stage *N*. (N=1, 2, 3, 4)

$\boldsymbol{Q_{stage\ N\ (B\ to\ A)}}$: The amount of charge transferred from Point B to Point A (labeled in the **Fig. 2A**) at Stage *N*. (N=1, 2, 3, 4)

$\boldsymbol{Q_{stage\ N}}$: The absolute value of the amount of charge transferred at Stage *N*.

II) Charge transfer calculation:

➢ **Initial stage**（Start from Stage 1: when the Electrode E3 touches the Electrode E_L）

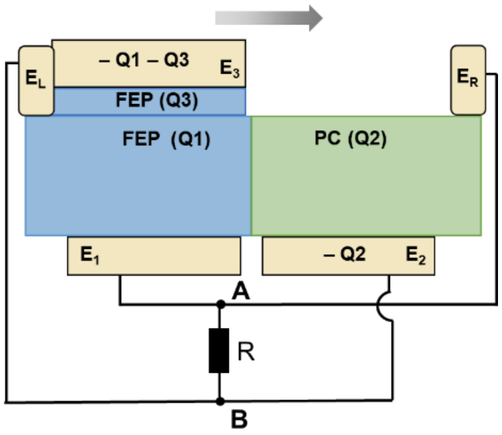

Supplementary Figure 33. Schematic of OCT-TENG operated at Stage 1.

At stage 1,

$$Q_{E2\_stage\ 1} = -Q_2 \tag{1}$$

$$Q_{E1\_stage\ 1} + Q_{E3\_stage\ 1} = -(Q_1 + Q_3) \tag{2}$$

$$\frac{Q_{E3\_stage\ 1}}{Q_{E1\_stage\ 1}} = \frac{C_3}{C_2} = \frac{\varepsilon_3 d_1}{\varepsilon_1 d_3} \tag{3}$$

$$Q_{E3\_stage\ 1} = -(Q_3 + Q_1)\frac{\varepsilon_3 d_1}{\varepsilon_3 d_1 + \varepsilon_1 d_3} \tag{4}$$

$$Q_{E1\_stage\ 1} = -(Q_3 + Q_1)\frac{\varepsilon_1 d_3}{\varepsilon_3 d_1 + \varepsilon_1 d_3} \tag{5}$$

When $\varepsilon_3 \approx \varepsilon_1$ and $d_1 \gg d_3$, **Eq. 4** and **Eq. 5** can be written as:

$$Q_{E3\_stage\ 1} = -Q_1 - Q_3 \tag{6}$$

and

$$Q_{E1\_stage\ 1} = 0 \tag{7}$$

➢ **Stage 2:** (The slider moves towards but before touching Electrode E_R)

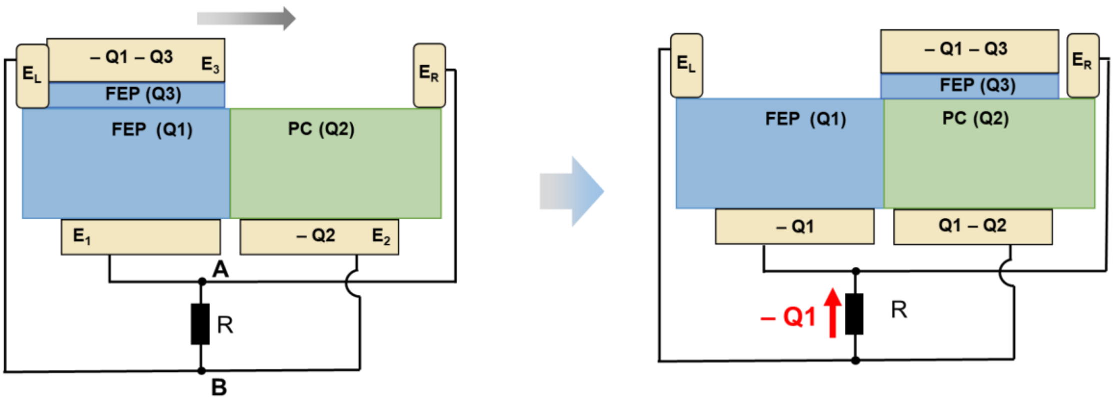

Supplementary Figure 34. Schematic of OCT-TENG operated at Stage 2.

At the end of stage 2,

$$Q_{E1\_stage\ 2} = -Q_1 \tag{8}$$

$$Q_{E3\_stage\ 2} = Q_{E3_{stage1}} = -(Q_3 + Q_1)\frac{\varepsilon_3 d_1}{\varepsilon_3 d_1 + \varepsilon_1 d_3} \tag{9}$$

$$Q_{E2\_stage\ 2} = -(Q_2 + Q_3 + Q_{E3\_stage\ 2}) = -(Q_2 + Q_3 - (Q_3 + Q_1)\frac{\varepsilon_3 d_1}{\varepsilon_3 d_1 + \varepsilon_1 d_3}) = -Q_2 + \frac{\varepsilon_1 d_3 Q_3 - \varepsilon_3 d_1 Q_1}{\varepsilon_3 d_1 + \varepsilon_1 d_3} \quad (10)$$

According to **Eq. 5** and **Eq. 8,** the charge transferred to Electrode E1 is:

$$Q_{stage\ 2\ (B\ to\ A)} = Q_{E1\_stage\ 2} - Q_{E1\_stage\ 1} = -Q_1 + (Q_3 + Q_1)\frac{\varepsilon_1 d_3}{\varepsilon_3 d_1 + \varepsilon_1 d_3} = \frac{\varepsilon_1 d_3 Q_3 - \varepsilon_3 d_1 Q_1}{\varepsilon_3 d_1 + \varepsilon_1 d_3} \quad (11)$$

According to **Eq. 1** and **Eq. 10**, the charge transferred to Electrode E1 is:

$$Q_{stage\ 2\ (A-B)} = Q_{E2_{stage2}} - Q_{E2_{stage1}} = -Q_2 - \left(-Q_2 + \frac{\varepsilon_1 d_3 Q_3 - \varepsilon_3 d_1 Q_1}{\varepsilon_3 d_1 + \varepsilon_1 d_3}\right) = -\frac{\varepsilon_1 d_3 Q_3 - \varepsilon_3 d_1 Q_1}{\varepsilon_3 d_1 + \varepsilon_1 d_3} \quad (12)$$

According to **Eq. 11** and **Eq. 12**, we get:

$$Q_{stage\ 2\ (A-B)} = -Q_{stage\ 2\ (B-A)} = \frac{\varepsilon_1 d_3 Q_3 - \varepsilon_3 d_1 Q_1}{\varepsilon_3 d_1 + \varepsilon_1 d_3} \quad (13)$$

When $\varepsilon_3 \approx \varepsilon_1$ and $d_1 \gg d_3$,

$$Q_{stage\ 2\ (A-B)} = Q_1 \quad (14)$$

When Q_1 <0, the amount of transferred charge is $Q_{stage\ 2} = -Q_1$, and the electric current direction is from B to A.

➢ **Stage 3** (The slider touching Electrode ER)

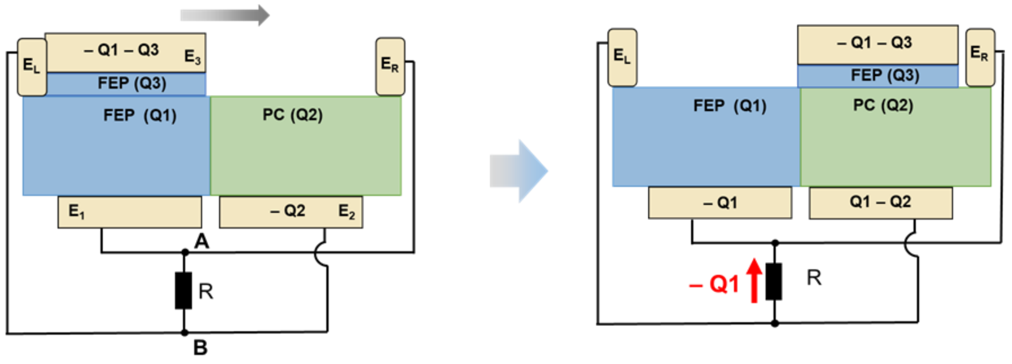

Supplementary Figure 35. Schematic of OCT-TENG operated at Stage 3.

At stage 3,

$$Q_{E1\_stage\ 3} = -Q_1 \tag{15}$$

$$Q_{E3\_stage\ 3} + Q_{E2\_stage\ 3} = -(Q_3 + Q_2) \tag{16}$$

$$\frac{Q_{E3\_stage\ 3}}{Q_{E2\_stage\ 3}} = \frac{C_3}{C_2} = \frac{\varepsilon_3 d_2}{\varepsilon_2 d_3} \tag{17}$$

$$Q_{E3\_stage\ 3} = -(Q_3 + Q_2)\frac{\varepsilon_3 d_2}{\varepsilon_3 d_2 + \varepsilon_2 d_3} \tag{18}$$

$$Q_{E2\_stage\ 3} = -(Q_3 + Q_2)\frac{\varepsilon_2 d_3}{\varepsilon_3 d_2 + \varepsilon_2 d_3} \tag{19}$$

According **Eq. 9** and **Eq. 18,** the charge transferred to Electrode E3 at Stage 3 is:

$$\begin{aligned} Q_{Stage\ 3\ (B\ to\ A)} &= Q_{E3_{stage}3} - Q_{E3_{stage}2} = \\ -(Q_3 + Q_2)\frac{\varepsilon_3 d_2}{\varepsilon_3 d_2 + \varepsilon_2 d_3} &- \left[-(Q_3 + Q_1)\frac{\varepsilon_3 d_1}{\varepsilon_3 d_1 + \varepsilon_1 d_3}\right] \\ = -\left[(Q_3 + Q_2)\frac{\varepsilon_3 d_2}{\varepsilon_3 d_2 + \varepsilon_2 d_3} \right. &\left. - (Q_3 + Q_1)\frac{\varepsilon_3 d_1}{\varepsilon_3 d_1 + \varepsilon_1 d_3}\right] \end{aligned} \tag{20}$$

According to **Eq. 10** and **Eq. 19**, at stage 3, the amount of charge transferred to Electrode E2 is

$$Q_{Stage\ 3\ (A\ to\ B)} = Q_{E2_{stage}3} - Q_{E2_{stage}2}$$

$$\begin{aligned} &= -(Q_3 + Q_2)\frac{\varepsilon_2 d_3}{\varepsilon_3 d_2 + \varepsilon_2 d_3} - [-(Q_2 + Q_3 - (Q_3 + Q_1)\frac{\varepsilon_3 d_1}{\varepsilon_3 d_1 + \varepsilon_1 d_3})] \\ &= (Q_3 + Q_2)\frac{\varepsilon_3 d_2}{\varepsilon_3 d_2 + \varepsilon_2 d_3} - (Q_3 + Q_1)\frac{\varepsilon_3 d_1}{\varepsilon_3 d_1 + \varepsilon_1 d_3} \end{aligned} \tag{21}$$

According to **Eq. 20** and **Eq. 21**, we get:

$$\begin{aligned} Q_{Stage\ 3\ (A\ to\ B)} &= -Q_{Stage\ 3\ (B\ to\ A)} \\ = (Q_3 + Q_2)\frac{\varepsilon_3 d_2}{\varepsilon_3 d_2 + \varepsilon_2 d_3} &- (Q_3 + Q_1)\frac{\varepsilon_3 d_1}{\varepsilon_3 d_1 + \varepsilon_1 d_3} \end{aligned} \tag{22}$$

When $\varepsilon_3 \approx \varepsilon_1$ and $d_1, d_2 \gg d_3$, **Eq. 22** can be simplified as

$$Q_{stage\ 3\ (A\ to\ B)} = Q_2 - Q_1 \tag{23}$$

When $Q_2 > Q_1$ (for instance, Q_2 is positive and Q_1 is negative), the amount of transferred charge is $Q_{stage\ 3} = Q_2 - Q_1$, and the electric current direction is from A to B.

➢ **Stage 4** (The slider moves towards but before touching Electrode E_L)

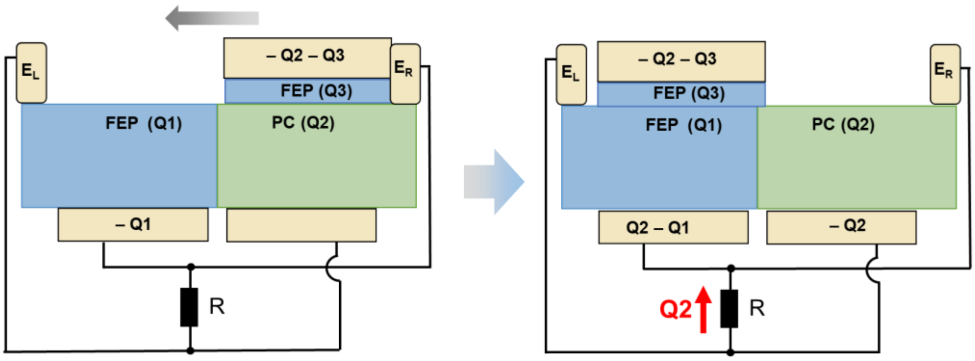

Supplementary Figure 36. Schematic of OCT-TENG operated at Stage 4.

At stage 4,

$$Q_{E2\_stage\ 4} = -Q_2 \tag{24}$$

$$Q_{E3\_stage\ 4} = Q_{E3\_stage\ 3} = -(Q_3 + Q_2)\frac{\varepsilon_3 d_2}{\varepsilon_3 d_2 + \varepsilon_2 d_3} \tag{25}$$

$$\begin{aligned} Q_{E1\_stage\ 4} &= -Q_{E3\_stage\ 4} - Q_1 - Q_3 \\ &= (Q_3 + Q_2)\frac{\varepsilon_3 d_2}{\varepsilon_3 d_2 + \varepsilon_2 d_3} - Q_1 - Q_3 \\ &= -Q_1 + Q_2\frac{Q_2\varepsilon_3 d_2 - Q_3\varepsilon_2 d_3}{\varepsilon_3 d_2 + \varepsilon_2 d_3} \end{aligned} \tag{26}$$

According to **Eq. 19** and **Eq. 24**, at stage 4, the amount of charge transferred to Electrode E2 is

$$\begin{aligned} Q_{stage\ 4\ (A\ to\ B)} &= Q_{E2\_stage\_4} - Q_{E2\_stage\_3} = -Q_2 + (Q_3 + Q_2)\frac{\varepsilon_2 d_3}{\varepsilon_3 d_2 + \varepsilon_2 d_3} \\ &= \frac{\varepsilon_2 d_3 Q_3 - \varepsilon_3 d_2 Q_2}{\varepsilon_3 d_2 + \varepsilon_2 d_3} \end{aligned} \tag{27}$$

According to **Eq. 15** and **Eq. 26**, at stage 4, the amount of charge in Electrode E1 is

$$\begin{aligned} Q_{tage\ 4\ (B\ to\ A)} &= Q_{E1_{stage}4} - Q_{E1_{stage}3} \\ &= -Q_1 + \frac{Q_2\varepsilon_3 d_2 - Q_3\varepsilon_2 d_3}{\varepsilon_3 d_2 + \varepsilon_2 d_3} - (-Q_1) \\ &= \frac{Q_2\varepsilon_3 d_2 - Q_3\varepsilon_2 d_3}{\varepsilon_3 d_2 + \varepsilon_2 d_3} \end{aligned} \tag{28}$$

According to **Eq. 27** and **Eq. 28**, we get:

$$Q_{Stage\ 4\ (A\ to\ B)} = -Q_{Stage\ 4\ (B\ to\ A)} = \frac{\varepsilon_2 d_3 Q_3 - \varepsilon_3 d_2 Q_2}{\varepsilon_3 d_2 + \varepsilon_2 d_3} \tag{29}$$

When $\varepsilon_3 \approx \varepsilon_1$ and $d_2 \gg d_3$,

$$Q_{stage\ 4\ (A\ to\ B)} = -Q_2 \tag{30}$$

When Q_2 >0, the amount of transferred charge is $Q_{stage\ 4} = Q_2$, and the electric current direction is from B to A.

➢ **Stage 1** (The slider touching Electrode E_L)

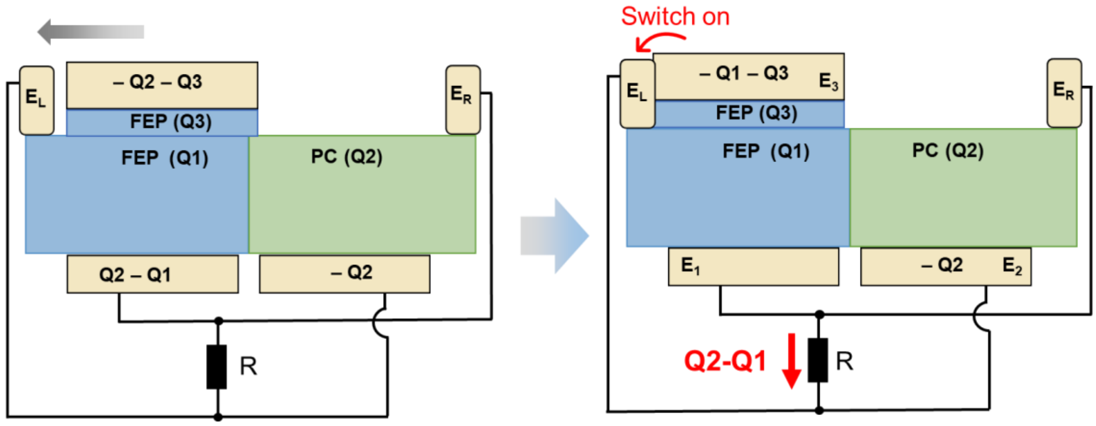

Supplementary Figure 37. Schematic of OCT-TENG operated at Stage 4.

At Stage 1,

$$Q_{E2\_stage\_1} = -Q_2 \tag{31}$$

$$Q_{E1\_stage\ 1} + Q_{E3\_stage\ 1} = -(Q_1 + Q_3) \tag{32}$$

$$\frac{Q_{E3\_stage\ 1}}{Q_{E1\_stage\ 1}} = \frac{C_3}{C_1} = \frac{\varepsilon_3 d_1}{\varepsilon_1 d_3} \tag{33}$$

$$Q_{E3\_stage\ 1} = -(Q_3 + Q_1)\frac{\varepsilon_3 d_1}{\varepsilon_3 d_1 + \varepsilon_1 d_3} \tag{34}$$

$$Q_{E1\_stage\ 1} = -(Q_3 + Q_1)\frac{\varepsilon_1 d_3}{\varepsilon_3 d_1 + \varepsilon_1 d_3} \tag{35}$$

According to **Eq. 25** and **Eq. 34**, at stage 1, the amount of charge transferred to Electrode E3 is

$$Q_{stage\ 4\ (A\ to\ B)} = Q_{E3\_stage\ 1} - Q_{E3\_stage\ 4}$$
$$= -(Q_3 + Q_1)\frac{\varepsilon_3 d_1}{\varepsilon_3 d_1 + \varepsilon_1 d_3} - [-(Q_3 + Q_2)\frac{\varepsilon_3 d_2}{\varepsilon_3 d_2 + \varepsilon_2 d_3}]$$
$$= (Q_3 + Q_2)\frac{\varepsilon_3 d_2}{\varepsilon_3 d_2 + \varepsilon_2 d_3} - (Q_3 + Q_1)\frac{\varepsilon_3 d_1}{\varepsilon_3 d_1 + \varepsilon_1 d_3} \tag{36}$$

According to **Eq. 26** and **Eq. 35**, at stage 1, the amount of charge transferred to

Electrode E1 is

$$Q_{stage\ 1(B\ to\ A)} = Q_{E1_{stage}1} - Q_{E1_{stage}4}$$

$$= -(Q_3 + Q_1)\frac{\varepsilon_1 d_3}{\varepsilon_3 d_1 + \varepsilon_1 d_3} - \left[(Q_3 + Q_2)\frac{\varepsilon_3 d_2}{\varepsilon_3 d_2 + \varepsilon_2 d_3} - Q_1 - Q_3\right]$$

$$= (Q_3 + Q_1)\frac{\varepsilon_3 d_1}{\varepsilon_3 d_1 + \varepsilon_1 d_3} - (Q_3 + Q_2)\frac{\varepsilon_3 d_2}{\varepsilon_3 d_2 + \varepsilon_2 d_3} \tag{37}$$

According to **Eq. 36** and **Eq. 37**, we get:

$$Q_{Stage\ 1\ (A\ to\ B)} = -Q_{Stage\ 4\ (B\ to\ A)}$$

$$= (Q_3 + Q_2)\frac{\varepsilon_3 d_2}{\varepsilon_3 d_2 + \varepsilon_2 d_3} - (Q_3 + Q_1)\frac{\varepsilon_3 d_1}{\varepsilon_3 d_1 + \varepsilon_1 d_3} \tag{38}$$

When $\varepsilon_3 \approx \varepsilon_1$ and $d_2 \gg d_3$,

$$Q_{stage\ 1\ (A\ to\ B)} = Q_2 - Q_1 \tag{39}$$

When Q_2 >0, the amount of transferred charge is $Q_{stage\ 1} = Q_2 - Q_1$, and the electric current direction is from A to B.

Reference:

1 Qin, H. *et al.* High energy storage efficiency triboelectric nanogenerators with unidirectional switches and passive power management circuits. *Advanced Functional Materials* **28**, 1805216 (2018).
