# Supplementary material for: Achieving ultrahigh instantaneous power density of 10 MW/m2 by leveraging the opposite-charge-enhanced transistor-like triboelectric nanogenerator (OCT-TENG)
Source: Nat Commun. 2021 Sep 15;12:5470. doi: 10.1038/s41467-021-25753-7 (PMC8443631; doi:10.1038/s41467-021-25753-7)
Supplement: Supplementary file 3 — Description of Additional Supplementary Files [file 41467_2021_25753_MOESM3_ESM.pdf]

Description of Supplementary Files

Supplementary Movie 1: Powering a 36W lamp using OCT-TENG

Supplementary Movie 2: Powering 180W lamps using OCT-TENG

Supplementary Movie 3: Wirelessly powering 825 LEDs using OCT-TENG

Supplementary Movie 4: Wirelessly powering 30W LEDs using OCT-TENG

Supplementary Movie 5: Powering a thermometer and a watch using OCT-TENG
